# Global sensing of the antigenic structure of herpes simplex virus gD using high-throughput array-based SPR imaging

**DOI:** 10.1371/journal.ppat.1006430

**Published:** 2017-06-14

**Authors:** Tina M. Cairns, Noah T. Ditto, Huan Lou, Benjamin D. Brooks, Doina Atanasiu, Roselyn J. Eisenberg, Gary H. Cohen

**Affiliations:** 1 Department of Microbiology, School of Dental Medicine, University of Pennsylvania, Philadelphia, Pennsylvania, United States of America; 2 Wasatch Microfluidics, Salt Lake City, Utah, United States of America; 3 Department of Pathobiology, School of Veterinary Medicine, University of Pennsylvania, Philadelphia, Pennsylvania, United States of America; Northwestern University, UNITED STATES

## Abstract

While HSV-2 typically causes genital lesions, HSV-1 is increasingly the cause of genital herpes. In addition, neonatal HSV infections are associated with a high rate of mortality and HSV-2 may increase the risk for HIV or Zika infections, reinforcing the need to develop an effective vaccine. In the GSK Herpevac trial, doubly sero-negative women were vaccinated with a truncated form of gD2 [gD2(284t)], then examined for anti-gD serum titers and clinical manifestations of disease. Surprisingly, few vaccinees were protected against genital HSV-2 but 86% were protected from genital HSV-1. These observations suggest that subtle differences in gD structure might influence a protective response. To better understand the antigenic structure of gD and how it impacts a protective response, we previously utilized several key anti-gD monoclonal antibodies (mAbs) to dissect epitopes in vaccinee sera. Several correlations were observed but the methodology limited the number of sera and mAbs that could be tested. Here, we used array-based surface plasmon imaging (SPRi) to simultaneously measure a larger number of protein-protein interactions. We carried out cross-competition or “epitope binning” studies with 39 anti-gD mAbs and four soluble forms of gD, including a form [gD2(285t)] that resembles the Herpevac antigen. The results from these experiments allowed us to organize the mAbs into four epitope communities. Notably, relationships within and between communities differed depending on the form of gD, and off-rate analysis suggested differences in mAb-gD avidity depending on the gD serotype and length. Together, these results show that gD1 and gD2 differ in their structural topography. Consistent with the Herpevac results, several mAbs that bind both gD1 and gD2 neutralize only HSV-1. Thus, this technology provides new insights into the antigenic structure of gD and provides a rationale as to how vaccination with a gD2 subunit may lead to protection from HSV-1 infection.

## Author summary

Understanding the complex steps of herpes simplex virus (HSV) entry into susceptible cells is key for developing an effective vaccine to prevent clinical disease and control recurrences of this important human pathogen. For either serotype (HSV-1 or HSV-2), it is the interaction of glycoprotein D (gD) with receptor that initiates the cascade of events to begin infection. Importantly, gD stimulates high titers of virus-neutralizing antibodies that predict its importance in a protective response. Many HSV vaccines under development center around gD. In one recent clinical trial, subjects vaccinated with gD2 were protected against HSV-1 genital infection but not HSV-2, leading us to address how type specificity affects the antigenic topography of gD. Using a large panel of monoclonal antibodies (mAbs), we employed a new high-throughput, array-based approach to organize the mAbs into communities. Relationships within and between communities differed depending on the isotype and length of gD, suggesting significant differences in the epitope topography. We found that a subset of mAbs bound both gD1 and gD2 yet only neutralized HSV-1, possibly accounting for the anomalous results of the clinical trial. This new technology has proven to yield significant insights about how different regions of gD contribute to immune responses.

## Introduction

Herpes simplex virus (HSV) is an important human pathogen. It first infects epithelial cells, then spreads to the peripheral nervous system to establish a lifelong, latent infection. Periodic reactivation from latently infected neurons leads to the production of infectious virus, which is released (shed) at mucosal sites. HSV-1 typically causes oral cold sores, while HSV-2 typically causes genital lesions. In the United States, 47% of the population is seropositive for HSV-1 and 16% for HSV-2, making HSV-2 the second most common sexually transmitted infection [1]. Several studies have provided evidence that prior genital herpes infection increases the risk of acquiring sexually transmitted HIV [2, 3]; emerging evidence suggests that HSV-2 may also increase the risk for Zika virus infection during pregnancy [4]. HSV can be transmitted from mother to child at birth and, although rare, neonatal HSV infections are associated with a high rate of mortality [5].

Understanding the complex steps of HSV entry is key for developing effective vaccines that prevent viral infection. Four viral glycoproteins are necessary for HSV entry into host cells: gD, gB, and the heterodimer gH/gL [6–8]. The current model of HSV entry evokes a step-wise process [6, 7, 9]. Initially, gD binds to one of its cellular receptors (nectin-1, herpesvirus entry mediator [HVEM], or 3-*O*-sulfotransferase heparin sulfate) [8, 10], which activates gD through conformational changes that presumably allow it to interact with and activate gH/gL, which in turn upregulates gB into a fusogenic state [11–13]. The class III fusion protein gB [14, 15] then facilitates fusion of the viral and cellular membranes and the subsequent entry of the viral capsid into the host cell [16–19].

Thus, conformational changes in gD triggered by receptor binding are critical to the entry process and identifying these changes will provide essential insight for vaccine strategies. Multiple crystal structures of gD have been solved including co-crystals with HVEM and nectin-1 [20–22], and a co-crystal with an antibody that blocks the binding of these receptors [23]. These structures revealed that the C-terminus of the ectodomain normally occludes the binding site for the receptor nectin-1 and prevents formation of the N-terminal loop needed for HVEM binding [20, 21, 24]. Several of the crystal structures reveal that receptor binding results in conformational changes to the ectodomain C-terminus. It is postulated that this altered and activated form of gD triggers downstream events leading to virus-cell fusion and entry [9, 11, 25]. Consistent with this postulate is that when a soluble form of gD is truncated at residue 285 and lacks the C-terminal tail, it bound to both receptors with 100-fold greater affinity than a form of gD [gD(306t)] with an intact C-terminal tail [26]. This increase in affinity of the gD/receptor interaction is due to a higher kinetic association rate constant or “on-rate” [27, 28]. Thus, for either receptor to bind to full-length gD1 or gD2, the ectodomain C-terminus must change conformation and be displaced. Interestingly, a mutant gD protein engineered to limit movement of the C-terminal tail was able to bind both HVEM and nectin-1, yet failed to trigger cell-cell fusion or complement a gD-null virus [29]. The phenotype of this mutant separated the receptor binding function from downstream, post-receptor-binding events also mediated by gD.

Many neutralizing monoclonal antibodies (mAbs) against gD block the binding of one or both receptors [23, 27, 30, 31]. However, among those neutralizing mAbs that do not block receptor binding [32], we hypothesized that they may interfere with the next step of entry, the activation of gH/gL by gD [9, 11, 25]. Previous studies have shown that mAbs that block receptor binding map to one “face’ of the three dimensional structure of gD, while those that are involved in subsequent steps map to the opposite “face” [32]. In addition, a linear stretch of gD termed the “pro-fusion domain” (residues 261–305) [33] that functions downstream of receptor binding has been implicated as a possible site of gD-gHgL interaction [25, 34, 35]. These regions of gD are clearly targets for vaccines and therapeutics.

In 2002, the National Institutes of Health and GlaxoSmithKlein undertook a clinical trial (Herpevac Trial for Women) where the main goal was to protect women against genital herpes disease [36]. Although the vaccine candidate was a subunit form of HSV-2 gD, the vaccine was only effective against HSV-1 [36]. Consistent with this unexpected outcome, clinical strains of HSV-1 were more sensitive than those of HSV-2 to neutralization by sera from gD2-vaccinated individuals [37]. Among the gD2 vaccine trial participants, serum antibodies against gD2, but not cell-mediated immunity, correlated with protection against HSV-1 genital disease [38, 39]. Indeed, the strongest correlates of neutralization were shown to be the overall gD-specific IgG content, as well as the combined response to several key gD epitopes [40]. Furthermore, the antibody response to the gD vaccine mimics that of naturally infected individuals [41, 42].

Given the relevance of gD-specific antibodies to virus neutralization and protection from disease, our goal in the current study was to generate a comprehensive examination of the antigenic structure of gD using an epitope binning (antibody competition) and mapping analysis that employs high-throughput array-based surface plasmon resonance imaging (SPRi) [43–45]. This assay provides a major technological advance in the tools to study the topological antigenic structure of the viral proteins. Here, we compared the antigenic structures of four isotypes of gD which differed in virus type (HSV-1 and HSV-2) and gD ectodomain length (285t and 306t). Each gD isotype was screened against a large panel of anti-gD mAbs that have broad epitopic coverage. Neutralization assays and the kinetic analysis of gD off-rates were also conducted to allow a structural interpretation of the cross-competition data. Although gD1 and gD2 share 85% amino acid identity overall, we detected antigenic differences by analyzing both mAb competition and network plots as well as gD off-rates of the mAbs. One striking difference was the diminished binding response of a group of highly neutralizing, receptor-blocking mAbs (group Ib) to gD(306t). We propose that this altered binding is due to the obstruction of these epitopes by the longer gD ectodomain tail found in gD(306t). This observation correlates with the reduced affinity of HVEM and nectin-1 to both serotypes of gD(306t) [27, 28, 46, 47], as the receptor binding sites and group Ib epitopes overlap [23, 48, 49]. Of importance, we identified several anti-gD mAbs that although generated against gD2, exhibited type-1 specific neutralization. These data mimicked the results from the Herpevac Trial and may explain in part why vaccinees were only protected from HSV-1 genital disease.

In all, we have demonstrated the ability to use a large panel of mAbs to globally sense the antigenic structure of four different forms of gD. We used these data to pin point those regions on the four forms of gD that were similar and those that differed. Lastly, we created a topographic epitope map of gD that was then transposed onto the gD crystal structure and emphasized its multiple “faces.” These findings will help facilitate our understanding of HSV neutralization with an eye towards the improvement of vaccine and/or therapeutic design.

## Results

Previously, we had grouped anti-gD mAbs into an antigenic dendrogram or “tree” [32, 40, 50]. The mAb groupings were based on a variety of properties, including the nature of the epitope (conformation-dependent vs. linear) and HSV type specificity [51]. Where possible, residues recognized by the mAb were defined using various mutant forms of gD, e.g. deletion and point mutants, as well as binding to synthetic peptides [52–57]. Competition assays were also carried out to further differentiate the epitope groups [54, 58, 59]. For example, while members of group I mutually competed with one another for binding to gD, applying other criteria such as residues for mAb-resistant mutant viruses (mars) and receptor blocking led to their being sorted into group Ia or group Ib [30, 53, 59]. Still, due to throughput limitations, some mAbs remained unclassified.

**Fig 1:**
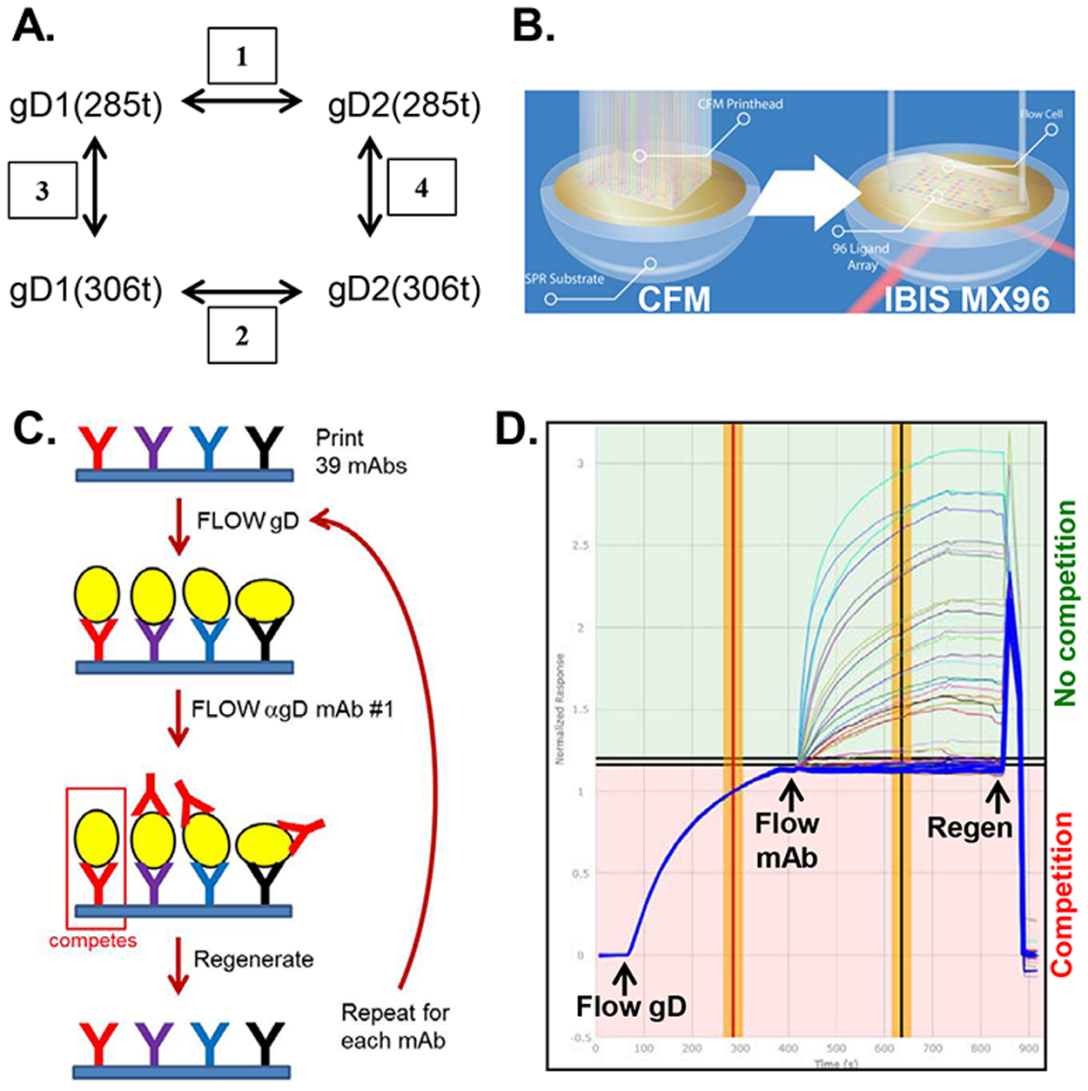
Examination of gD isoforms using a high-throughput, array-based SPRi protocol. (A) Comparisons made between different forms of gD in this study. gD was compared between types (gD1 vs. gD2, boxes 1 & 2), and between length of gD (306t vs. 285t, boxes 3 & 4). (B) To create discrete protein arrays and measure the binding across multiple mAbs in parallel, the Wasatch CFM was used, followed by detection of the injected sample in an IBIS MX96 biosensor. SPRi was used to detect protein binding interactions across the array. (C) Graphical representation of the Wasatch CFM/SPRi protocol. The individual anti-gD mAbs are printed to distinct spots on the biosensor chip (shown as distinctly colored Ys). The antigen (gD, yellow ovals) is then flowed across the chip surface and captured by each mAb. Next, the first anti-gD mAb (analyte) is flowed across the chip surface and its binding capacity measured. Alternatively, to test mAb blocking of nectin-1 binding, nectin is flowed across the chip. Lastly, the chip surface is regenerated back down to the level of the printed mAbs and the cycle is repeated for each individual mAb to be tested. (D) An overlay of individual sensorgrams for 56 cycles on a single printed mAb (LP2) for classical sandwich epitope binning. Responses are normalized at the end of the gD capture step. The thick blue curves denote gD-only controls (no mAb flowed). Responses in the pink (lower) section of the sensorgram above the blue curves are from competing mAbs; responses in the green (upper) section belong to non-competing mAbs. The red/yellow vertical bar indicates where the response units (RU, y-axis) for the printed mAb was derived for gD binding; the black/yellow vertical bar is where RUs for the flowed mAb (analyte) were taken.

Here, using high-throughput epitope binning, we have reorganized and refined our tree (S1 Fig). This approach is designed to characterize the blocking profiles of mAbs in both directions (as analyte and ligand), thus allowing for the validation of relationships within and across our original groups [44, 45]. A major advantage is that cross-competition can easily be carried out between large numbers of mAbs (analytes) and ligands (e.g. gD) with small sample volumes. Hence, our goal was to characterize and expand upon the antigenic and structural relationships among mAb epitopes between four forms of gD: gD1(285t), gD2(285t), gD1(306t), and gD2(306t) (Fig 1A), which represent the two viral forms of HSV gD (type-1 and type-2), as well as two different lengths of the gD ectodomain (285 and 306 residues). Both forms of gD2 have been studied in various vaccine trials [60–62] and any differences detected will be directly relevant to vaccine design.

**Fig 2:**
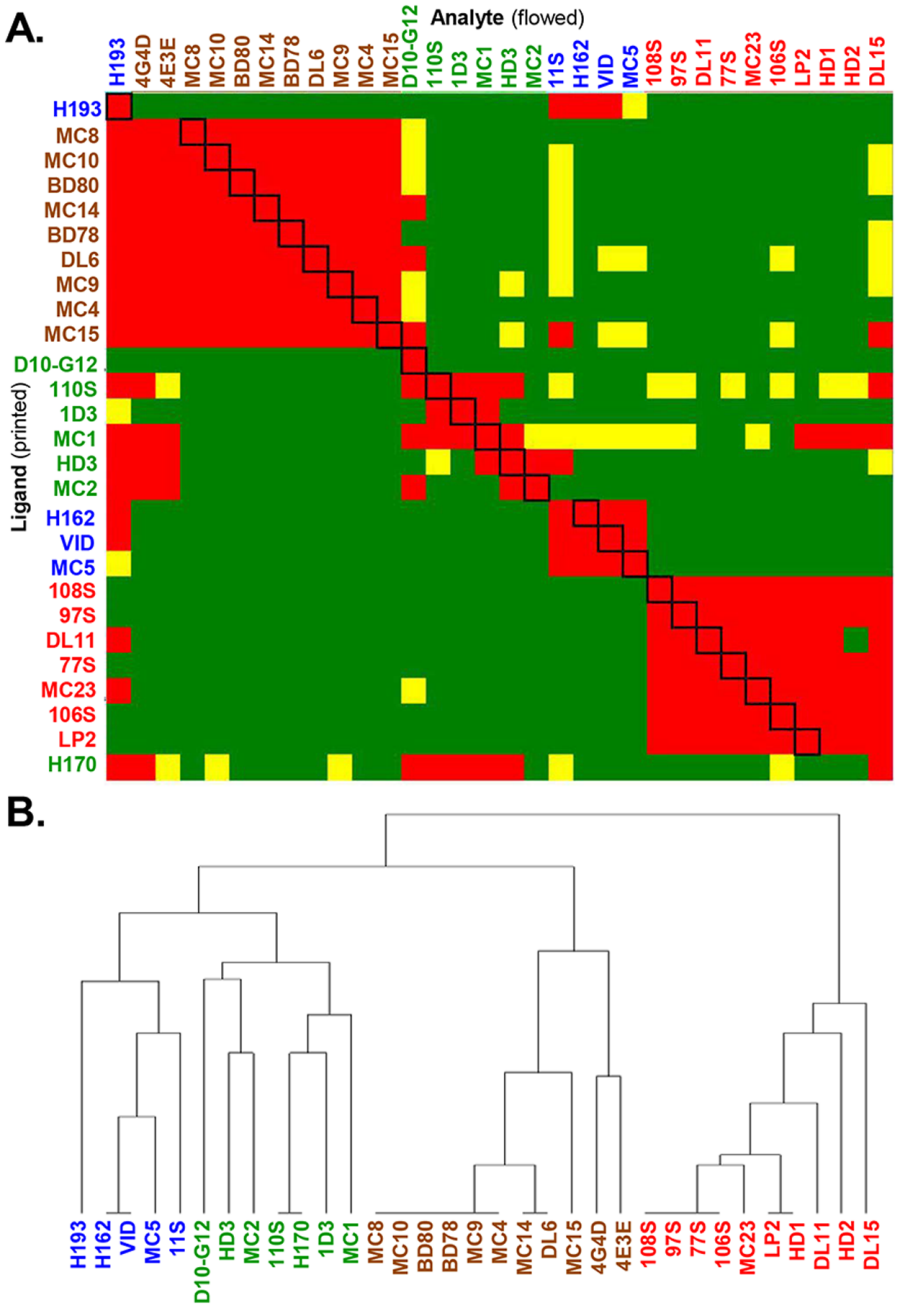
Binning of mAbs against gD2(285t). (A) Heat map. Red boxes denote competition between the ligand mAb and the analyte mAb. The black boxes outlined diagonally show self-self mAb competition. Green boxes indicate no competition between mAbs and a high level of binding of the analyte mAb onto gD as bound by the ligand mAb. Yellow boxes show a lower level of binding for the analyte, but the mAbs are still scored as not competing. Excluded from analysis: ligand mAbs that bound ≤10 response units of gD; mAbs that blocked >80% of other mAbs. For gD2(285t), 33 of our 39 mAbs are represented. Of those mAbs that are missing from the heat map, three are type-1 specific (A18, 3D5, 45S), two require residues 286–306 (AP7, 12S), and one bound ≤10 RU of gD (11B3AG). (B) Combined dendrogram. Horozontal lines at the base of the denogram indicate distinct bins of mAbs; they have identical competition profiles (example, H162 and VID). mAb names for both (A) and (B) are colored according to community groupings as shown in Fig 3B.

A panel of 39 mAbs (IgG) chosen for this study (Table 1) was covalently arrayed on the SPR sensor chip surface using the Wasatch CFM, and then placed into the IBIS MX96 biosensor (Fig 1B). One of the four forms of gD (Fig 1A) was then injected across the chip surface, followed by each of the 39 mAbs injected in series (Fig 1C) [44]. After being tested in this pair-wise, combinatorial manner, the mAbs were arranged by the software into bins to reflect competition vs. no competition [43–45]. Our assay, using 39 mAbs and the 4 forms of gD, generated approximately 6,000 sensorgrams (Fig 1D) which are then algorithmically analyzed to generate a heat map (Fig 2A) and dendrogram (Fig 2B) detailing the relationships of the mAb bins for each form of gD tested. Fig 2 shows the heat map and dendrogram generated against one of the four gD constructs, gD2(285t). In the heat map (Fig 2A), red boxes denote competition between the ligand mAb and the analyte mAb. Green boxes indicate no competition between mAbs, while yellow boxes show a lower level of binding for the analyte mAb, but the mAbs are still scored as not competing. Heat maps/dendrograms generated with the same panel of mAbs against the other forms of gD, gD1(285t), gD1(306t), and gD2(306t), are shown in Supplemental S2–S4 Figs.

**Table 1:** Properties of anti-gD mAbs.

Group	mAb	gD binding	Epitope residues	Plaque Assay IgG (μg/mL) for 50% neutralization of:	
				HSV-1 (KOS)	HSV-2 (333)
Ia	**HD1**	TC	216	4 +/- 1.4	4.7 +/- 1.8
	**HD2**	TC	ND	4 +/- 1.4	6.5 +/- 2.1
	**LP2**	TC	216	2.6 +/- 3.3	2.7 +/- 1.1
	**MC23**	TC	213, 216	1.6^a^	0.26^a^
	DL15*	TC	ND	3.2 +/- 4	NN
Ib	**DL11**	TC	38, 132, 140, 222–224	0.004^a^	0.31^a^
	77S*	TC	38, 222–224	0.1 +/- 0.1	1.4 +/- 1.6
	97S*	TC	38, 222–224	0.9 +/- 0.1	1.8 +/- 2.3
	106S*	TC	38, 222–224	2.3 +/- 1	2.1 +/- 1.9
	108S*	TC	38, 222–224	0.6 +/- 0.5	0.4 +/- 0.5
IIa	**MC4**	TC	262–272	NN^a^	NN^a^
	**MC8**	TC	262–272	NN^a^	NN^a^
	**MC9**	TC	262–272	NN^a^	NN^a^
	**MC10**	TC	262–272	NN^a^	NN^a^
	**MC14**	TC	262–272	NN^a^	NN^a^
	**MC15**	TC	262–272	NN^a^	NN^a^
	**BD78**	TC	262–272	21 +/- 12.7	12 +/- 4.2
	**BD80**	TC	262–272	19 +/- 6.6	9 +/- 0
IIb	**DL6**	TC	272–279	NN^a^	NN^a^
IIc	4E3E*	TC	ND	2.3 +/- 2.4	5 +/- 5.6
	4G4D*	TC	ND	3 +/- 0	16.2 +/- 18
III	**VID**	TC	54	3^b^	13^b^
	**11S**	TC	ND	5.3 +/- 2.6	NN
	3D5*	T1S	ND	6.7 +/- 1.1	NN
	**MC5**	TC	75–79	3.1^a^	6.2^a^
	H162*	TC	ND	20.7 +/- 27.2	7.3 +/- 5.1
	H193*	TC	ND	21.7 +/- 25.8	3.9 +/- 3
IV	**45S**	T1S	ND	21.3 +/- 17.2	NN
	D10-G12*	TC	ND	2.5 +/- 1.9	NN
VII	**110S**	TC	1–29	1.8 +/- 2.4	5 +/- 5.6
	**1D3**	TC	10–20	0.39^a^	6.2^a^
	**MC1**	TC	10–20	NN	NN
	**H170**	TC	1–23	5.9 +/- 1.6	20.7 +/- 14.1
X	**HD3**	TC	ND	4 +/- 0.7	22.5 +/- 10.6
XII	**AP7**	TC	25, 27, 294	24.7 +/- 22.7	NN
	12S*	TC	ND	2.5 +/- 1.4	NN
XVII	11B3AG*	TC	ND	0.9 +/- 0.6	NN
	A18*	T1S	246	12.5 +/- 7.8	NN
	**MC2**	T2S	246	NN^a^	0.78^a^

NN, non-neutralizing (>25 μg/mL); TC, type common; T1S, type-1 specific; T2S, type-2 specific; ND, not determined; +/1 indicates standard deviation (minimum of 2 experiments).

* newly characterized

^a^ as reported in Lazear et al. [32]

^b^ see Materials and methods

**Fig 3:**
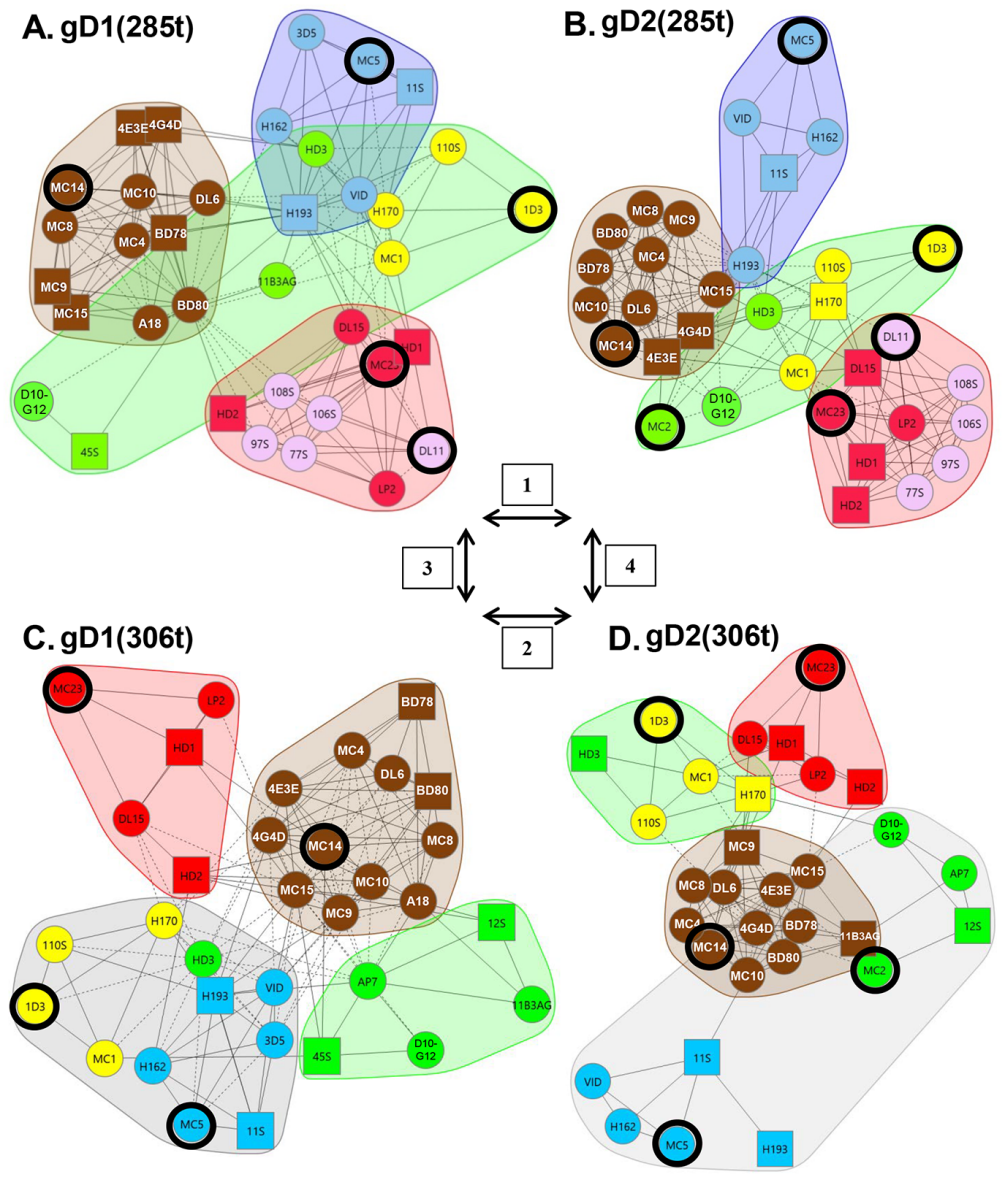
Community mAb plots. For each form of gD, the mAbs were divided into four communities and colored accordingly. (A) gD1(285t). (B) gD2(285t). (C) gD1(306t). (D) gD2(306t). Antibody names in a circle indicate that competition was measured as both a ligand and an analyte; antibody names in a square indicate that competition was measured in one direction only, as either a ligand or an analyte. Solid connecting lines specify that competition between the two mAbs was seen as both a ligand and analyte for each. However, dashed connecting lines identify that the competition between mAbs was seen in one direction only (example, MC1 (ligand) blocked LP2 (analyte) binding, but LP2 (ligand) did not block MC1 (analyte) binding on gD2(285t). Yellow spheres/squares highlight group VII mAbs (N-terminal, linear epitopes), while pink spheres track the group Ib mAbs; mAb groups are shown in Table 1. Black rings highlight key mAbs (1D3, DL11, MC2, MC5, MC14, MC23) used in previous studies. Arrows and boxed numbers denote comparisons between the 4 gD forms as described in Fig 1A. Antibody 11B3AG was omitted from the gD2(285t) mapping (B) due to low gD binding.

**Fig 4:**
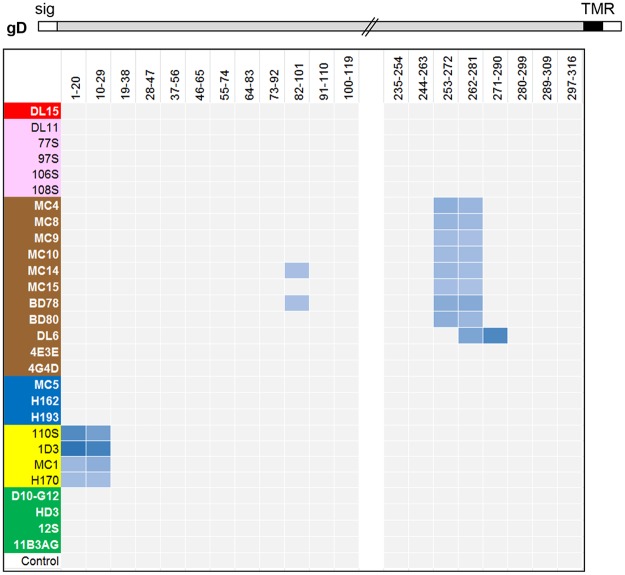
Map representing the binding of mAbs to an array of overlapping peptides corresponding to the gD ectodomain. Antibody names are shown on the y-axis and colored according to community/group as shown in Fig 3. Peptides are denoted by gD residue numbers and are shown on the x-axis. Binding of mAb to peptide is shown as a blue box. A stick drawing of gD is shown at the top. Hatched lines indicate a break in the amino acid sequence between residues 120–234 where no mAbs bound. Sig, signal sequence; TMR, transmembrane region.

Using Fig 1A as a guide for our studies, we compared the four forms of gD. Grouping of mAbs by CFM-SPRi relies on the underlying binary assignment of competitive/ noncompetitive behaviors generated from the binning experiment (Fig 2A). From these assignments, mAbs with similar behaviors are grouped into a network plot, descriptive of which epitopes are engaged. The result is a graphical representation of the heat map where mAbs are sorted into “communities” (Fig 3). For each form of gD, mAbs were sorted into four communities distinguishable by color: red, green, blue, and brown (Fig 3). The arrangement of the mAbs within and between communities depended on the extent of competition. In each community plot, solid lines that connect mAbs signify that the two mAbs compete in both directions (i.e., both as ligands and analytes). Dashed lines that connect mAbs indicate competition in one direction only (e.g., for mAbs that could not be analyzed as ligands (see Materials and methods), competition data was gathered only as analytes). The distance between mAbs as depicted in the community plots connotes relatedness of mAbs: the spatially closer mAbs are, the more they share similarity in competitive nature. In essence, a community plot abstracts out nuanced data, allowing for identification of broader relationships between mAbs. For example, mAbs in the same bin (which competed with identical sets of mAbs) are now grouped into a larger community, and this community is situated near others according to competitive relatedness. Thus, the communities provide a picture of the epitope landscape of gD. Below, we will compare the results for each of the network (community) plots formed by the four forms of gD. To further analyze our unclassified mAbs, we used the CFM to print a library of overlapping, biotinylated gD2 peptides [40] and then screened them for mAb binding (see Materials and methods). As negative controls, we included two previously characterized mAbs that bind conformation-dependent epitopes (DL11, MC5). All mAbs in Table 1 previously known to bind peptides were also included as positive controls. Data is summarized in Fig 4 and details of mapping will be discussed below for each community.

### Comparison of gD1(285t) vs. gD2(285t)

We first asked whether the panel of anti-gD mAbs could distinguish between gD1(285t) and gD2(285t) (Fig 3A and 3B). Both formed nearly identical mAb network plots, as the four communities (brown, red, blue, green) are situated in a spatially similar way. However, differences were detected in the arrangement of mAbs both in their respective location within a given community and their distance from each other, suggesting subtle differences in structure. Below, we will detail the mAb arrangement within and across each of the four communities.

#### Brown community

Except for mAb A18, which is type-1 specific [63], the brown community of gD1(285t) contains all the same mAbs as that of gD2(285t) (Fig 3A and 3B). Most members of this community were originally defined as type-common, non-neutralizing mAbs that recognize linear epitopes between residues 262–279 and assigned to group II (S1 Fig, Table 1) [32, 50]. In addition, both brown communities contain two previously unclassified mAbs, 4E3E and 4G4D.

From the peptide mapping data we found that with the exception of 4E3E, 4G4D, A18, and DL6, all of the mAbs in the brown community bound to two peptides near the ectodomain C-terminus, one that spanned residues 253–272 and the other spanning residues 262–281 (Fig 4). The overlap between these adjacent peptides narrowed the epitope to residues 262–272, corresponding to our original mapping of these mAbs into group II (S1 Fig, Table 1) [54]. DL6 bound peptides 262–281 and 271–290 (Fig 4), placing the epitopes between 272–280 and again confirming previous data [54]. These results emphasize the power of the CFM-SPRi technology to screen large numbers of mAbs against many peptides and yield reliable data. Curiously, mAbs MC14 and BD78 bound to a second, separate linear region (residues 82–101), something not seen previously, an observation that may be due to sequence similarity between peptides. Antibodies A18, 4E3E, and 4G4D did not bind peptides (Fig 4), suggesting that they bind to conformation-dependent epitopes. However, their strong competition with the linear C-terminal mAbs facilitated their inclusion in the brown community (Fig 3A and 3B) and group II (Table 1, S1 Fig).

#### Green community

This community consists of seven mAbs that recognized both gD1(285t) and gD2(285t) and two type-specific mAbs, 45S (gD1) and MC2 (gD2) (Fig 3A and 3B). MC2, a major neutralizing mAb [32], competes with brown community members 4E3E and 4G4D, positioning these two close to the green community on gD2(285t) (Fig 3B). Antibodies MC2, 4E3E, and 4G4D are examples of “gateway” mAbs that compete with member mAbs from other communities. These results demonstrate how the network plots give nuanced data regarding the antigenic structure of gD.

Three previously unclassified mAbs were sorted into the green community: HD3, 11B3AG, and D10-G12 (Fig 3A and 3B). These three mAbs did not bind gD peptides (Fig 4) and were characterized as binding conformation-dependent epitopes. Among the green community mAbs were a cluster of type-common linear mAbs (1D3, 110S, H170, MC1) previously shown to recognize epitopes within the first 25 residues of gD (group VII, Table 1; Fig 4) [27, 30, 53]. We have colored these mAbs yellow so their location can be easily tracked within and between the communities of the four forms of gD.

#### Blue community

Two previously unclassified mAbs, H162 and H193, were sorted into the blue community on both gD1(285t) and gD2(285t) (Fig 3A and 3B). These two mAbs bind conformation-dependent epitopes and not peptides (Fig 4). A third mAb, 3D5, was found to be type-1 specific for gD binding yet competed strongly for gD binding with the other type-common mAbs in the blue community (Fig 3A). A major, type-common, neutralizing mAb, MC5 [32], was the only member mAb in group XVI of our original tree [32]. Here, MC5 was sorted into the blue community for both gD1(285t) and gD2(285t) (Fig 3A and 3B) along with two members of our original group III mAbs, VID and 11S (Table 1). The strong competition between all members of the blue community and the close proximity of mutations on the gD 3D structure that negate both MC5 and VID binding [32, 50] lead us to refocus our concept of MC5. MC5, 3D5, H162, and H193 were placed into group III along with VID and 11S (Table 1, S1 Fig).

#### Red community

In our original mAb tree, group I consists of a series of type-common, virus-neutralizing mAbs that recognize a major functional region overlapping the nectin-1 binding site [27, 30, 31, 49, 50, 64]. Although all group I mAbs compete with each other for gD binding, mapping data with mutant proteins and viruses distinguished DL11 from HD1, HD2, LP2, and MC23, thereby splitting them into two subgroups, Ia and Ib [31, 49, 50, 53, 56, 59]. Not surprisingly, both group Ia and Ib were sorted into the red communities of both gD1(285t) and gD2(285t) (Fig 3A and 3B). The two red communities also included five previously uncharacterized mAbs (DL15, 77S, 97S, 106S, and 108S). These five mAbs bound conformation-dependent epitopes (Fig 4). To discriminate between group Ia and Ib, the mAbs were screened for binding against several mutant gD proteins. Four of the mAbs (77S, 97S, 106S, and 108S) failed to bind to gD mutants Y38A and Δ222–224 (S5 Fig), which is consistent with other members of group Ib [31, 49]. To distinguish between the two subgroups, we colored mAbs from group Ia red and group Ib pink on the community maps (Fig 3). As with the other communities, there were several mAbs that cross-competed between communities, displaying a continuum of competition. For example, DL15 and MC23 have multiple lines of competition to the blue and green communities on both gD1(285t) and gD2(285t) (Fig 3A and 3B). Interestingly, several other group I mAbs displayed cross-community competition on either gD1(285t) or gD2(285t) (Fig 3A and 3B), suggesting that the two forms of gD exhibit subtle topographic differences.

### Comparison of gD1(306t) vs. gD2(306t)

Next, the antigenic landscape of gD1 was compared to that of gD2 in the background of gD(306t), a longer form of the gD ectodomain. Unlike the overall similarities between types on gD(285t), there were striking differences both within and between communities on gD(306t).

#### Brown community

The member mAbs of the brown community were identical between gD1(306t) and gD2(306t) with one exception, 11B3AG, which sorted to the brown community for gD2(306t) and the green community for gD1(306t) (Fig 3C and 3D). This difference was apparent from the additional competitive lines between 11B3AG and some members of the brown community on gD2(306t) that were not seen on gD1(306t). Also, mAbs in the brown community of gD2(306t) were more tightly arranged, indicating that they shared more closely related competition profiles. This was also observed between the brown communities of gD1(285t) and gD2(285t) (Fig 3A and 3B), suggesting that these mAbs discriminate a subtle type-difference (gD1 vs gD2) between gD forms.

#### Green and blue communities

The composition and arrangement of mAbs in the blue and green communities diverge markedly between gD1(306t) and gD2(306t). On the gD1(306t) background, mAbs 11B3AG, 45S, D10-G12, AP7, and 12S were placed in the green community (Fig 3C). Antibodies AP7 and 12S bind gD(306t) but not gD(285t) [24, 26, 56, 57]. Also on gD1(306t), mAbs from the group III (blue) and group VII (yellow) were assigned to the same community. However, these two groups were spatially separated onto opposite sides of the community (Fig 3C). Antibody HD3, which was in the green community on gD(285t), was now situated between the group III and VII mAbs. Thus, the data delineated a unique community on gD1(306t) that included mAbs from group III (blue) and group VII (yellow), and HD3 (green).

Unlike the situation for gD1(306t), group III (blue) and group VII (yellow) mAbs sorted to different communities on gD2(306t) (Fig 3D). Group VII (yellow) was once again sorted into the green community [as it was on gD(285t)] alongside mAb HD3. However, another unique community was formed from group III (blue) and previously green community mAbs AP7, 12S, MC2, and D10-G12 (Fig 3D). These two sets of mAbs did not compete for gD2(306t) binding, yet because of the numbers of competition events and types of perceived cross-community competitions they were sorted into a single community. This distinction was borne out by the extreme spatial separation between the two halves of this community. The existence of this unique community was a by-product of the tight competition networks that the other three communities (red, brown, and green) exhibited on gD2(306t).

#### Red community

The red communities on gD1(306t) and gD2(306t) were both comprised exclusively of group Ia mAbs (Fig 3C and 3D). The only differences between these two red communities were 1) spacing between the mAbs (reflecting competitive differences), and 2) placement among the other three communities. On gD2(306t), the red community was in close proximity to the green community; on gD1(306t) the red and green communities were on opposite ends of the network plot, highlighting a possible topographic distinction. Most striking for the red communities of both gD1(306t) and gD2(306t) was the absence of all pink group Ib mAbs from the network plot (Fig 3A, 3B vs 3C and 3D) due to a striking reduction in the binding of group Ib mAbs to gD(306t) (see below).

### Comparison of gD(285t) to gD(306t)

**Fig 5:**
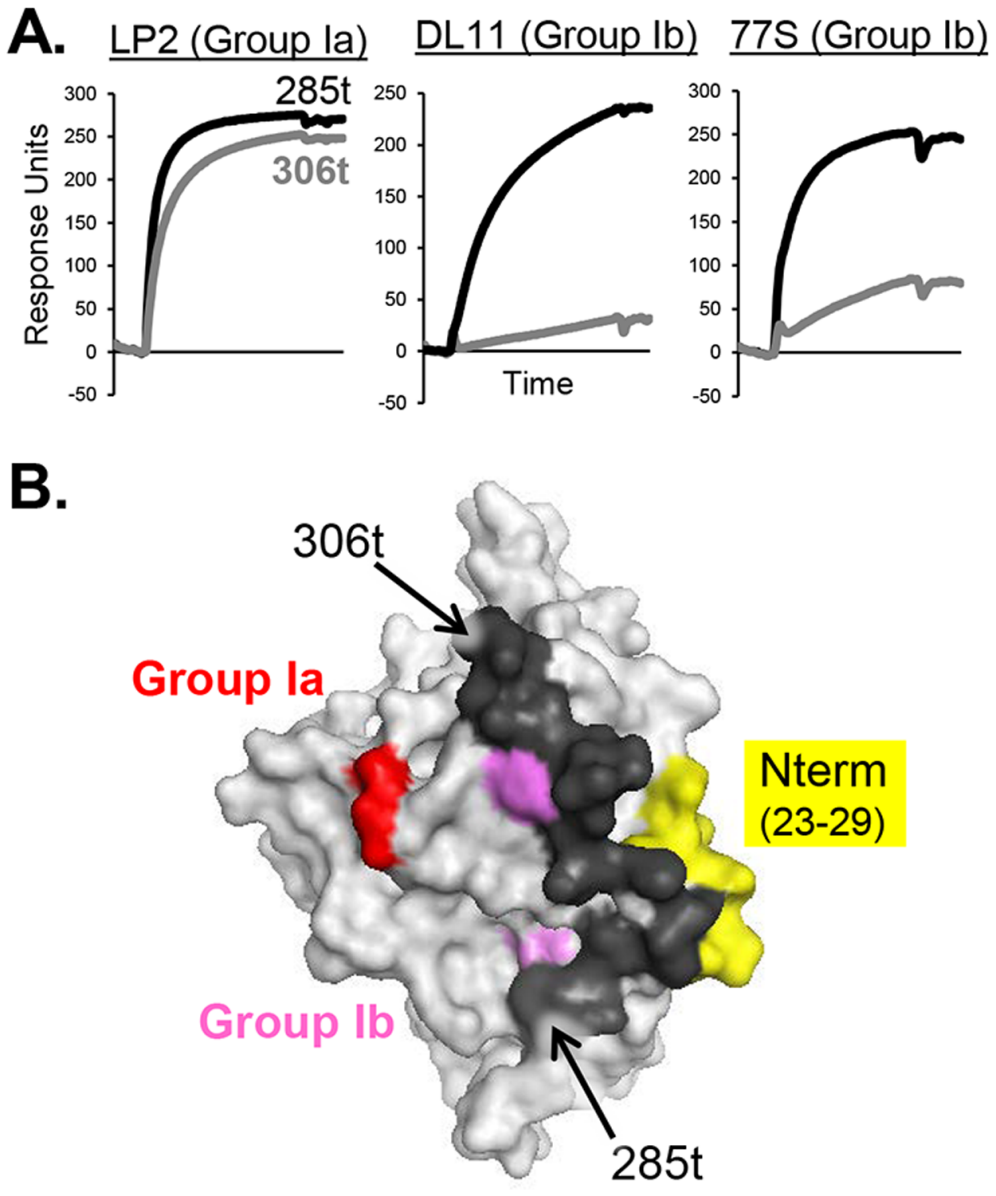
The gD ectodomain tail (residues 285–306, black) obscures the group Ib, but not group Ia, epitopes. (A) Binding of group I mAbs to gD(285t) (black curve) vs. gD(306t) (grey curve) via BIAcore. Group Ia is represented by mAb LP2, while group Ib is represented by mAbs DL11 and 77S. (B) Surface representation of the gD crystal structure is shown in grey (PDB 2C36). gD residues implicated in group Ib mAb binding (38, 132, 140, 222–224) are colored pink, while those for group Ia (213, 216) are colored red. The gD N-terminus, which is the site of group VII binding, is colored yellow.

Finally, this approach also showed that the community maps of gD(285t) (Fig 3A and 3B) differ significantly from that of gD(306t) (Fig 3C and 3D). Most striking was the absence of all pink group Ib mAbs from the gD(306t) maps (Fig 3C and 3D). Unlike group Ia mAbs, which bound equally well to gD(285t) and gD(306t), group Ib mAbs bound to gD(306t) poorly (Fig 5A). Therefore, gD binding values (response units) were too low to generate competition data for gD(306t). Most likely, it is the additional residues on the gD(306t) C-terminus (amino acids 286–306) that interfere with the binding of the pink group Ib mAbs, as these epitopes lie directly under this 21 amino acid C-terminal ectodomain tail (Fig 5B) [23, 24, 49]. Another prominent difference between gD(285t) and gD(306t) was the positioning of the yellow group VII mAbs (Fig 3) from within the green community on gD1(285t) (Fig 3A) to a community containing blue group III mAbs on gD1(306t) (Fig 3C). This shift might reflect an effect of the flexible C-terminal residues in gD(306t) on the equally flexible N-terminal residues [20, 25]. In support of this concept, the epitope for AP7 involves residues at both the N- and C-termini of gD(306t) [24, 26, 56, 57, 59]. Interestingly, apart from mAb 11B3AG, the brown community was unchanged between all four gD forms (Fig 3).

**Fig 6:**
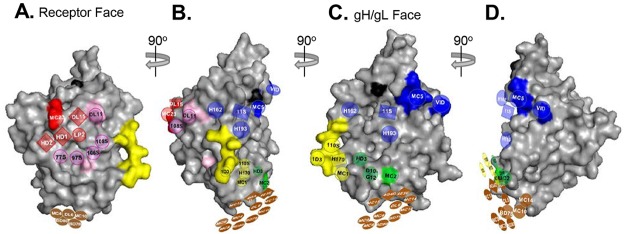
Overlaying the four gD mAb communities onto the gD 3D structure. Surface representation of the gD crystal structure is shown in grey (PDB 2C36). Black surface residues indicate glycosylation sites as a point of reference. Antibody-resistant mutations for individual mAbs (Table 1) were used to position the four colored mAb communities (red, brown, green, and blue) from Fig 3B onto the structure. These mutations are similarly colored on the surface of the gD structure to convey points of orientation. The communities for gD2(285t) were used so as to include group Ib mAbs and MC2. The gD1(306t) structure (the only published structure solved past amino acid 259) was chosen as our model to provide a structural basis for the brown community, which is positioned on residues 262–279. The gD structure is rotated 90° from (A) to (B), (B) to (C), and (C) to (D).

Combining our new community information with the known epitopes and functions of each mAb we were able to position each community onto the three-dimensional structure of gD to obtain a working model (Fig 6). This model will be explained in detail in the Discussion, as it gives us a topological antigenic map of gD for the first time.

### Analysis of gD-mAb off-rates to discriminate between gD forms

**Fig 7:**
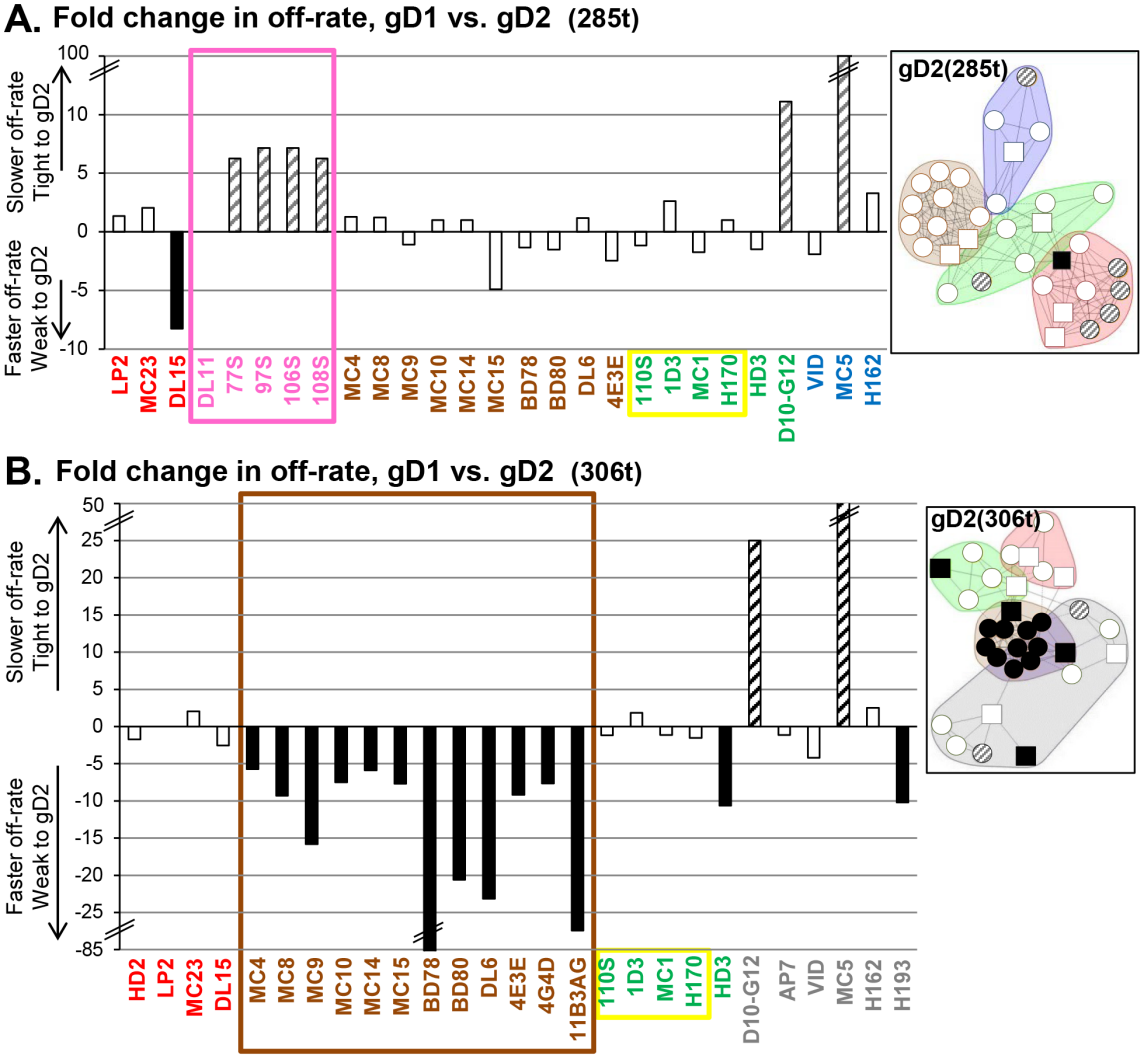
Comparison of fold changes in off-rate for gD as a function of sub-type (type-1 vs. type-2). Fold change was calculated from ratio of gD2 off-rate over the gD1 off-rate. gD1 vs. gD2 was tested in the context of both gD(285t) (A) and gD(306t) (B). Hash marks denote a break in the y-axis. Disassociation rates were determined as described in Materials and Methods; mAbs not listed above did not yield rate data. Bars are shaded to highlight greater than 5-fold change in rates. Black bars = faster off-rate for gD2 and hatched bars = slower off-rate for gD2, as compared to gD1. A lensed community map displaying the affected mAbs is shown to the right of each graph. Since the numbers in the graphs reflect an increase or decrease in off-rate on gD2, the mAbs were lensed on their respective forms of gD2. Antibody names are color-coded by community on either gD2(285t) (A) or gD2(306t) (B). A box is drawn around mAbs to highlight changes that cluster to one community or group.

To generate binning data, only final response levels were utilized. However, the real-time nature of these experiments permits investigation of association and dissociation rates of mAbs towards antigens. Using the same set of data from the binning analysis, samples were evaluated for sensorgram curvature as a measure of dissociation rates for gD against the immobilized (printed) mAbs (Table 2). In this way, we were able to compare the stability of the mAb-gD interaction for all four forms of gD (Fig 1A) as ratios between pairs of gDs, specifically the fold difference. Most of the mAbs against gD did not discriminate between gD1 and gD2 (ratio of 1, Fig 7), indicating that the avidity of binding was equivalent. However, in three instances the gD off-rates were different, highlighting structural differences between these two gD types.

**Table 2:** Dissociation rates (koff) for gD against immobilized anti-gD mAbs.

Group	mAb	gD1(306t)	gD1(285t)	gD2(306t)	gD2(285t)
**Ia**	**HD2**	5.70E-05	NA	9.90E-05	8.76E-04
	**LP2**	1.00E-05	1.35E-05	1.00E-05	1.00E-05
	**MC23**	2.22E-04	1.66E-03	1.09E-04	8.10E-04
	**DL15**	7.65E-04	3.54E-03	1.96E-03	2.92E-02
**Ib**	**DL11**	1.00E-05	1.00E-05	NA	1.00E-05
	**77S**	5.14E-04	6.67E-04	3.75E-04	1.07E-04
	**97S**	1.00E-05	7.24E-05	1.00E-05	1.00E-05
	**106S**	2.59E-05	7.34E-05	1.00E-05	1.00E-05
	**108S**	4.29E-05	6.28E-05	5.68E-05	1.00E-05
**IIa**	**MC4**	1.08E-04	7.85E-04	6.17E-04	6.21E-04
	**MC8**	8.50E-05	8.29E-04	7.90E-04	6.73E-04
	**MC9**	6.10E-05	7.93E-04	9.64E-04	8.74E-04
	**MC10**	9.60E-05	7.38E-04	7.21E-04	7.33E-04
	**MC14**	1.10E-04	6.75E-04	6.47E-04	6.71E-04
	**MC15**	1.35E-04	2.77E-04	1.04E-03	1.36E-03
	**BD78**	1.00E-05	2.72E-04	8.41E-04	3.66E-04
	**BD80**	5.00E-05	2.73E-04	1.03E-03	4.16E-04
**IIb**	**DL6**	1.56E-05	4.69E-04	3.61E-04	4.00E-04
**IIc**	**4E3E**	1.06E-04	6.40E-04	9.71E-04	1.58E-03
	**4G4D**	1.30E-04	NA	9.94E-04	NA
**III**	**VID**	6.70E-05	1.43E-04	2.80E-04	2.73E-04
	**11S**	6.72E-04	NA	NA	NA
	**3D5**	7.29E-04	4.65E-04	NA	NA
	**MC5**	5.87E-04	1.49E-03	1.00E-05	1.00E-05
	**H162**	2.71E-04	2.90E-04	1.08E-04	8.76E-05
	**H193**	3.30E-05	NA	3.37E-04	NA
**IV**	**45S**	7.01E-03	1.87E-02	NA	NA
	**D10-G12**	2.27E-04	4.48E-04	1.00E-05	3.90E-05
**VII**	**110S**	9.06E-04	1.79E-03	1.06E-03	2.07E-03
	**1D3**	5.95E-05	1.47E-04	3.22E-05	5.54E-05
	**MC1**	3.66E-04	5.79E-04	4.15E-04	1.00E-03
	**H170**	2.97E-04	3.12E-04	4.48E-04	3.09E-04
**X**	**HD3**	1.12E-04	3.23E-04	1.19E-03	4.81E-04
**XII**	**AP7**	6.07E-04	NA	6.90E-04	NA
	**12S**	1.00E-05	NA	NA	NA
**XVII**	**11B3AG**	3.28E-05	1.22E-03	9.00E-04	NA
	**A18**	8.90E-05	3.50E-04	NA	NA
	**MC2**	NA	NA	3.09E-04	2.90E-04

In the epitope binning experiments, a single, fixed concentration of the gD antigen was injected across the mAb surfaces, limiting rate fitting to dissociation only.

NA, not available

First, mAbs MC5 and D10-G12 showed the greatest change in binding between gD1 and gD2; in the background of 285t, gD2 bound with10-fold higher affinity to D10-G12 and almost 100-fold higher affinity to MC5 (Fig 7A). This difference in binding was independent of size, i.e. from 285t to 306t (Fig 7B); for gD(306t), gD2 bound D10-G12 with 25-fold higher affinity and MC5 with 50-fold higher affinity than gD1.

Second, red community mAbs detected a change in structure between gD1(285t) and gD2(285t) (Fig 7A). These mAbs all map to the group Ib (pink; 77S, 97S, 106S, and 108S) (Fig 1B) and exhibited a slower gD off-rate; DL11 was the outlier as it was the same for both isotypes of gD. For group Ia (red), only mAb DL15 had a gD off-rate change >5-fold; gD1 bound to DL15 8-fold better than gD2 (Table 2). We highlighted this clustering of off-rate phenotypes by coloring the community plots by off-rate (Fig 7A, right side). Interestingly, no changes in off-rates were found within the red community when gD was in its longer form, 306t (Fig 7B).

Third, mAbs in the brown community exhibited a faster gD2(306t) off-rate than gD1(306t) (Fig 7B). This clustering indicates that there is a significant change in gD2 structure as compared to gD1 at the ectodomain C-terminus, where the brown community epitopes lie. One possibility is that there exists a difference in the association of the C-terminal tail with the gD core between gD1 and gD2, which in turn affects the brown community epitopes. Although we published the structure of gD1(306t) by using a disulfide-bonded dimer (cysteine added at amino acid 307) [24], a similar structure has not been solved for gD2(306t). However, there is no difference between the off-rates of gD1(285t) and gD2(285t) when bound by brown community mAbs, suggesting that the lone type-specific amino acid difference within linear epitopes in this community (gD residue 269) is not a factor.

**Fig 8:**
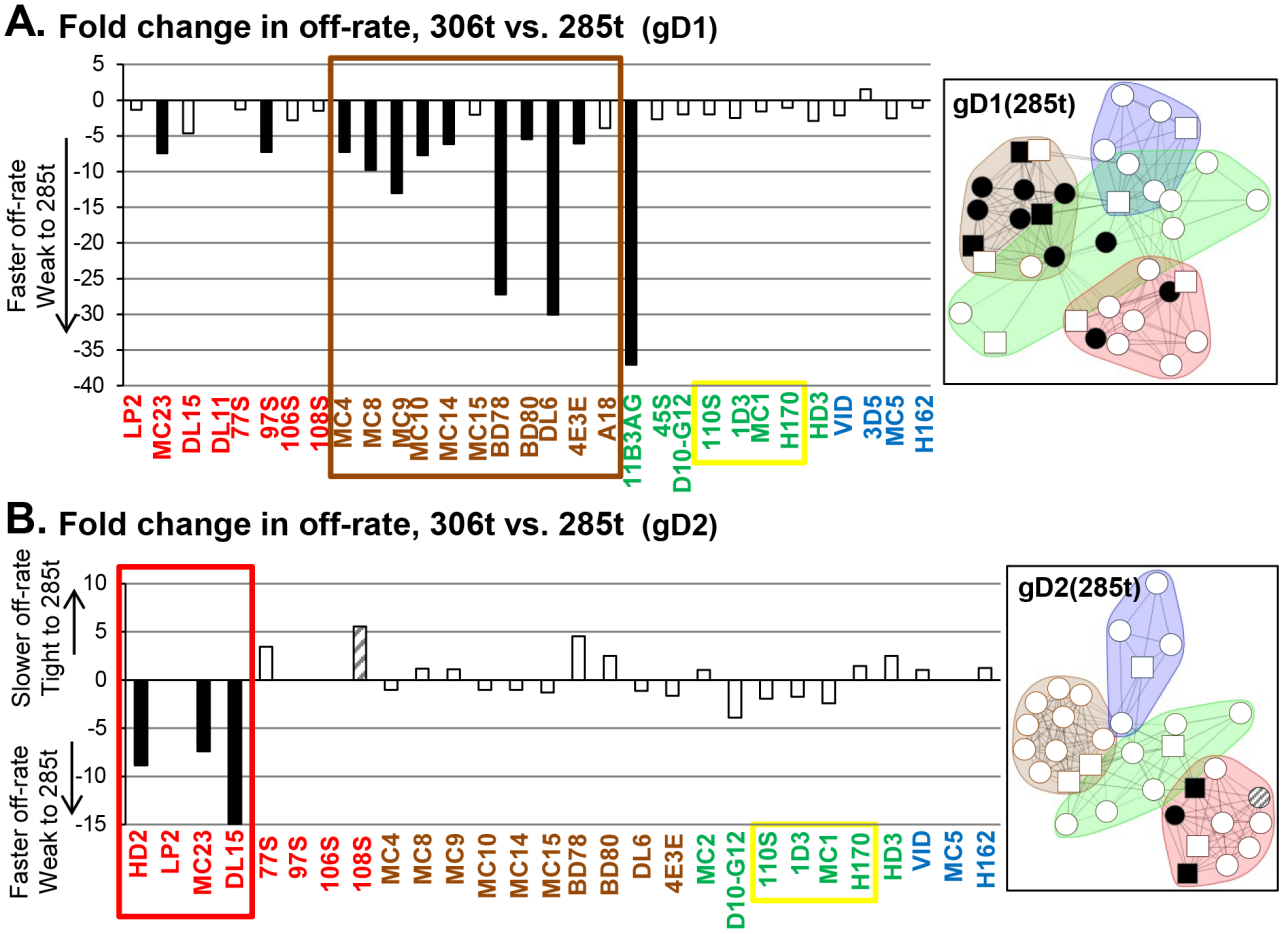
Comparison of fold changes in off-rate for gD as a function of protein length (306t vs. 285t). Fold change is calculated from ratio of 285t off-rate over the 306t off-rate. Protein length was tested in the context of both gD1 (A) and gD2 (B). Bars are colored to highlight greater than 5-fold change in rates as in Fig 7. Black bars = faster off-rate for 285t and hatched bars = slower off-rate for 285t, as compared to 306t. A lensed community map displaying the affected mAbs is shown to the right of each graph. Since the numbers in the graphs reflect an increase or decrease in off-rate on 285t, the mAbs were lensed on their respective forms of gD(285t). mAb names are color-coded by community on either gD1(285t) (A) or gD2(285t) (B).

A comparison of the off-rates between different gD lengths revealed several interesting findings. First, unlike what was seen for gD isotype (Fig 7), there were no changes in off-rate between gD(306t) and gD(285t) when bound to either MC5 or D10-G12 (Fig 8A and 8B). For these two mAbs, HSV type was important but not gD length. Second, in the brown community, several mAbs exhibited faster gD off-rates on gD1(285t) than gD1(306t) (Fig 8A, right side). This was not observed when we compared gD2(285t) with gD2(306t) (Fig 8B). Third, in a type-2 background, the only changes in gD off-rate were seen within the red community (Fig 8B). Interestingly, all off-rate changes between gD(285t) and gD(306t) were under 40-fold (Fig 8).

Lastly, the gD off-rates for yellow group VII mAbs (1D3, 110S, H170, and MC1) are unchanged across all 4 comparisons (Figs 7 and 8, yellow box), suggesting that the N-terminus is not affected by subtype or ectodomain length. Overall, the grouping of mAbs determined by changes in gD off-rate agrees with our groupings via mAb competition. However, the kinetic data provide additional information about these four proteins, allowing their further differentiation.

### Antibodies that have type-common binding can have HSV-1 specific neutralization

**Fig 9:**
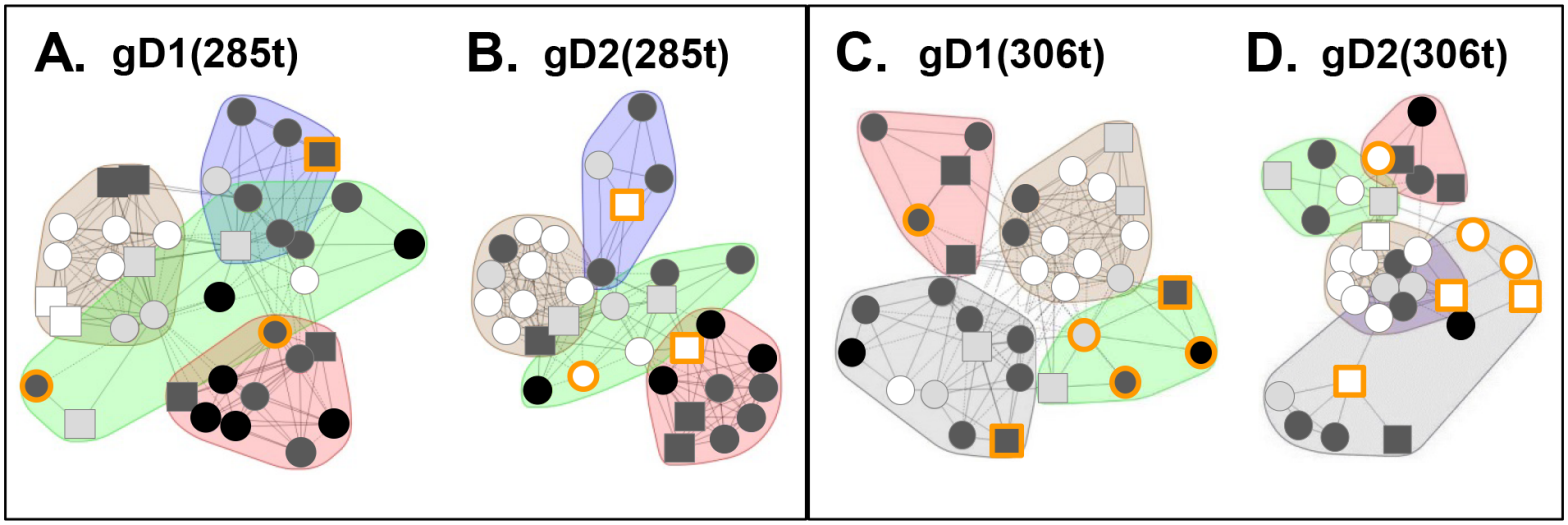
The virus neutralization activity of each mAb, depicted as a colored view of each network plot. Network plots for each of the four gD forms are the same as shown in Fig 3. (A) gD1(285t). (B) gD2(285t). (C) gD1(306t). (D) gD2(306t). Neutralization is lensed as a gradient from black to white, with black representing the highest level of activity and white representing no activity. The amount of mAb needed to neutralize 50% of plaques (mg/mL IgG) in a standard plaque reduction assay is listed in Table 1. Antibodies that bind both gD1 and gD2 but only neutralize HSV-1 are outlined in orange: 11S, D10-G12, DL15 for gD(285t) and 11S,D10-G12, DL15, AP7, 12S, 11B3AG for gD(306t).

Since mAb competition and gD off-rates analyses revealed antigenic changes between the four forms of gD, we asked if these changes correlated with virus neutralization. Each mAb was tested for neutralization against HSV-1 (KOS) and HSV-2 (333) using a standard 50% plaque reduction assay (Table 1). Then, each community plot was shaded by the approximate levels of virus neutralization by each mAb (shaded white to black, Fig 9). For gD1(285t) (Fig 9A), all four communities contained neutralizing mAbs. When compared to gD2(285t), three mAbs that neutralize HSV-1 fail to neutralize HSV-2 (Fig 9B, circled orange). These type-common mAbs (DL15, 11S, and D10-G12) bind well to both gD1 and gD2 yet only neutralize HSV-1 (Table 1). For gD(306t), we again see several mAbs that neutralize HSV-1 but not HSV-2 (Fig 9C and 9D); these include not only DL15, 11S, and D10-G12, but also 11B3AG and 12S. Additionally, mAb AP7 neutralizes HSV-1 weakly (with a 50% neutralizing IgG concentration just below the 25 μg/mL cut-off) and does not neutralize HSV-2. Both AP7 and 12S bind to gD(306t) but not gD(285t), suggesting an important type-1 specific, functional property lies in this region. When we examine the neutralization data in Table 1, three classes of type-common gD binding, neutralizing mAbs are observed: 1) mAbs that neutralize both HSV-1 and HSV-2; 2) mAbs that neutralize HSV-1 much better than HSV-2, i.e. DL11, which neutralizes HSV-1 76-fold better than HSV-2 [32]; and 3) mAbs that neutralize HSV-1 but not HSV-2.

To summarize, community mapping suggests that gD1 and gD2 are antigenically different in context of 306t but not 285t. However, merging the binning data with gD off-rate data reveals differences in the way certain mAbs bind gD1(285t) vs. gD2(285t). Likewise, mAbs that bind both gD1 and gD2 but have type-specific functions (e.g., neutralize HSV-1 but not HSV-2) suggest potential structural differences between gD1 and gD2.

## Discussion

As the receptor binding protein for HSV-1 and HSV-2, gD plays a critical role in initiating the virus entry fusion cascade. Conformational changes to gD structure that result from binding any one of the three cellular receptors are key not only to virus entry, but are also important in generating critical targets for neutralizing antibodies mounted by the host to thwart this process. HSV-1 and HSV-2 gD are 85% identical in amino acid composition, and most mAbs, both human and murine, generated from immunization or infection are type-common in their ability to bind gD (Table 1). However, there are several examples of mAbs that either bind to or neutralize gD in a type-specific manner (Table 1). In addition, although gD1 and gD2 can be substituted for each other in a cell-cell fusion assay [65], it was recently found that fusion by HSV-2 glycoproteins occurs twice as fast as that achieved by HSV-1 glycoproteins, a difference partially controlled by the gD serotype [11]. Hence, as similar as gD1 and gD2 are in structure, there are tangible differences between the two.

To enhance our understanding of the antigenic structures of gD1 and gD2, we used a high-throughput quantitative SPR-based antibody competition approach to determine the relationships of multiple anti-gD mAbs and assign them into spatially contiguous communities. A major advantage of this technique is that we could examine multiple interactions of a group of 39 mAbs against gD, testing a comprehensive matrix of cross-competition between all 39 mAbs in an unbiased manner. This enabled us to define relationships of mAbs not only within our original groups but to link the branches of our mAb “tree” (S1 Fig). Thus, in an independent fashion the paratope of each mAb “senses” the surface topography of the protein, reports back the magnitude of the interaction thereby defining the terrain, and establishing the “geographical” location of each mAb in relation to multiple and often diverse mAbs. The results led to a composite global sensing of the antigenic structure of four different forms of gD. The WM-SPR software then organized the mAbs into four spatially-related communities, each representing a group of mAbs that recognized overlapping antigenic sites.

Using the “antigenic global sensing” concept described above, we compared the community clusters across the four different forms of gD: gD1(285t), gD2(285t), gD1(306t), and gD2(306t) (Fig 1A). In the background of gD(285t), the communities for all four forms were quite similar in content and arrangement, which was not surprising given the 85% homology of gD1 and gD2. However, our binning results revealed fine differences in the structures of the two proteins, consistent with the two proteins being non-identical. The differences were more striking for the longer forms of gD1 and gD2, i.e. gD(306t), implying that residues within the C-terminal tail between amino acids 285 and 306 have a major effect on the presentation of epitopes on other parts of the two glycoproteins. These residues are also key for gD-receptor activity [24, 26, 29] and constitute part of the gD “pro-fusion domain” [33].

### Aligning mAb communities onto the gD three-dimensional structure

Combining community information with the location and known functions of each mAb we oriented each community onto the three-dimensional structure of gD1(306t) [24] and assigned the location of the communities (based on known *mar* mutations) to obtain a working model (Fig 6). As an overlay, we used the communities (red, blue, green, and brown) from gD2(285t) (Fig 3B) to include group Ib (pink) mAbs, and to include type-2 specific neutralizing mAb MC2. Antibodies that compete with MC2 have been detected in individuals both immunized with a gD2 subunit and infected with HSV [40–42].

Fig 6 displays gD in four configurations, offset 90° from each other. The brown community, which is centered along a linear stretch of amino acids that wraps along the bottom of the molecule (residues 262–279), is visible on each gD configuration. The red community, which contains the group I mAbs (Table 1), is located on the receptor binding face of gD (Fig 6A)[23, 32, 49]. When gD is rotated 90°, some members of the red community are still visible, but now we also see members of green and blue communities (Fig 6B). This side separates the receptor binding and gH/gL binding faces of gD. Here, the green community is represented primarily by group VII (highlighted in yellow) mAbs that block HVEM binding. We can also visualize how members of the blue community (e.g. H162) may recognize residues close to the receptor binding face, e.g. the DL11 epitope (Fig 6B).

Rotating gD another 90° (Fig 6C), we arrive at the gH/gL binding face [6, 32]. This side of gD is located 180° from the receptor binding face and was defined by neutralizing mAbs MC2 and MC5 [32], which are sentinel mAbs of the green and blue communities, respectively. Rotating gD the final 90° reveals a side of gD that is almost devoid of known epitopes (Fig 6D). Besides MC5 and VID, whose *mar* mutations (residues 54, 75–79; Table 1) map to the upper left corner, and the brown community epitopes along the bottom of the molecule, this side of gD is relatively empty.

### Analysis of gD off-rates reveals subtle differences in gD structures

The epitope binning results reveal that the brown community is viewed as the “anchor” between the four different forms of gD. Except for mAb 11B3AG, which “flipped” between the brown and green communities depending on gD form (Fig 3), mAbs in the brown community formed a tight competition network that was relatively unchanged between all four forms of gD. However, the off-rate data with these mAbs revealed structural differences between gD1 and gD2 near the epitopes of the brown community (Fig 7B). Two other off-rate phenomena also pointed to differences in gD1 vs. gD2 structure: within the red community, a slower gD(285t) off-rate on the group Ib mAbs (Fig 7A), and within the blue community, a 10- to 100-fold slower gD off-rate on mAbs MC5 and D10-G12 (Fig 7A and 7B). These, subtle type-specific differences in mAb stability emphasize that although gD1 and gD2 are 85% identical [23] they diverge in their antigenic landscapes to affect how epitopes are presented.

Remarkably, the off-rate data show that the N-terminus of gD (yellow, group VII) is unaffected by the presence of the ectodomain tail or the isotype of gD (Figs 7 and 8). These linear epitopes reside between amino acids 1–23 (Fig 4), which are 91% identical between gD1 and gD2. These mAbs form a tight-knit cluster regardless of which of the four gD forms is being studied (Fig 3). Notably, this region is poorly immunogenic in humans vaccinated with gD2 [40] or naturally infected with HSV [41, 42].

### Type-common mAbs that neutralize only HSV-1

We identified several type-common mAbs that only neutralize HSV-1 (Table 3). Two of these mAbs (D10-G12, DL15) were generated against type-2 virus or a purified gD2 subunit (Table 3). Antibodies with this phenotype sort into different communities (Fig 3), evidence that the epitopes that specify this property are not limited to one specific region of gD. These type-common mAbs apparently recognize a similar epitope on gD1 and gD2, but distinguish fine differences in the structure-function of the proteins. Interestingly, we have yet to find the reverse occurrence (type-common binding, HSV-2 specific neutralization) (Table 1).

**Table 3:** Anti-gD mAbs that bind gD1 and gD2 yet only neutralize HSV-1.

mAb	Antigen used to generate mAb
11S	HSV-1
12S	HSV-1
11B3AG	HSV-1
D10-G12	HSV-2
DL15	purified gD2

Finding multiple mAbs with this phenotype might explain the anomalous result of the Herpevac trial [36]. The implication is that immunization with a subunit form of gD2 may induce high-titer, type-common antibodies in the host that appear by ELISA to be quantitatively satisfactory, but qualitatively (i.e., protection and virus neutralization) insufficient. Indeed, for individuals in the Herpevac trial, neutralizing titers were found to be significantly higher for HSV-1 than for HSV-2 [37]. Other potential mechanisms have been proposed to explain the trial results [37]. For example, neutralizing Abs may be blocked from binding to gD2 but not gD1 by neighboring glycoproteins such as gC or gE [66]. It is also possible that fewer gD molecules are present on the envelope of HSV-1 strains than HSV-2 strains, making HSV-1 easier to neutralize.

Another potential mechanism for the type-specific neutralization displayed by some Abs may lie with differences in association/disassociation rates between gD1 and gD2 (Table 2, Fig 7). Two mAbs, 11B3AG and DL15, have a faster dissociation constant for HSV-2 gD, which may in part explain the lack of HSV-2 neutralization. However, off-rates are clearly not the only factor, since other mAbs (e.g., 4E3E) that neutralize both HSV-1 and HSV-2 equally well also display faster off-rates (weaker binding) to gD2. In the case of DL11, whose gD off-rate remains unchanged between gD1 and gD2 yet neutralizes HSV-1 76-fold greater than HSV-2 (Table 1) [32], the gD on-rate may play a role instead.

### Movement of the gD ectodomain tail is necessary for both group Ib mAb and receptor binding

Previously, we found that both cellular receptors exhibit a greater affinity for the gD(285t) form than for gD(306t), presumably due to the absence of the ectodomain C-terminus [26–28]. In this study, we found that certain mAbs that block receptor binding (pink, group Ib) bound both forms of gD(285t) well yet bound poorly to the longer version of gD, gD(306t) (Fig 5A). All published crystal structures of gD are of derivations of gD(285t) or shorter, except where the C-terminal tail (residues 286–306) is locked via a disulfide bond [20–24]. These structures revealed that the normally flexible C-terminal tail occludes the binding site for nectin-1 and prevents formation of the N-terminal loop needed for HVEM binding [20, 24]. Consequently, for either receptor to bind to gD, the C-terminal ectodomain “tail” must be displaced [49]. Now, within the red community, the binding of those mAbs that bind well to gD(285t) and poorly to gD(306t) fit with our previous prediction that residues in the binding sites for nectin-1, HVEM, and group I mAbs overlap [49].

Recently, the crystal structure of gD bound to a Fab fragment of a human mAb, E317, was determined [23]. E317 binds in the same region as the red community mAbs and shares many of their phenotypes. The gD-E317 Fab crystal structure shows that the epitope footprint occupied by the Fab overlaps multiple residues in the gD-nectin binding domain. Likewise, gD residues that participate in gD-E317 binding also are critical in gD-HVEM binding [20], likely interfering with formation of the gD N-terminal hairpin required for HVEM binding [20]. However, a comparison of the crystal structures of gD-E317 and gD-nectin-1 [21, 23] reveals that while the E317 and nectin-1 binding regions overlap extensively, the E317 Fab adopts a different binding mode and its area of interaction with gD is approximately threefold larger [23]. We speculate that the red community mAbs may mimic receptor in that they bind and trigger the cascade of gD conformational changes required for fusion and entry [9], thereby offering a possible second mechanism for HSV neutralization. These results suggest that neutralization may occur via both steric (receptor-blocking) and non-steric mechanisms.

In summary, the high-throughput quantitative SPR-based antibody approach used here has refined our understanding of the antigenic structure of HSV gD. Moreover, the results show that structure-function differences between gD1 and gD2 likely influenced the anomalous outcome of employing gD2 to induce a protective immune response specific against HSV-2 [36, 37]. Our data suggest that the most successful gD based vaccines should stimulate humoral responses against each of the key steps played by gD and should also target as many epitopes as possible for neutralizing antibodies in the four communities.

## Materials and methods

### Cells and soluble proteins

African green monkey kidney (Vero) cells were grown in Dulbecco’s modified Eagle medium (DMEM) with 5% fetal bovine serum (FBS). HSV type-1 and type-2 gD(285t) and gD(306t) were produced from baculovirus-infected insect (Sf9) cells and purified using a DL6 immunosorbant column [26, 32, 67]. Additional gD1 soluble proteins used in this study were previously reported: C-terminal truncations 250t, 260t, 275t, and 316t [24, 26, 31]; deletion mutant Δ(222–224) [31]; point mutants Y38A, V231W, and W294A [24, 25, 49]; and insertion mutants ins34, ins126, and ins243 [56, 67].

### Antibodies

The following anti-gD mAbs were previously published: 1D3 [32, 68, 69]; DL6, DL11, DL15 [32, 51, 53, 54, 58]; MC1, MC2, MC4, MC5, MC8, MC9, MC10, MC14, MC15, MC23 [32, 70]; A18 [63]; AP7, LP2 (kindly provided by A. Minson and H. Browne) [59]; HD1, HD2, HD3, H162, H170, H193 [71, 72]; 11S, 12S, 45S, 77S, 97S, 106S, 108S, 110S [68, 73]; BD78, BD80 (kindly supplied by Becton Dickinson Co.) [32, 54]; and the human mAb VID [50, 56, 74]. However, mAbs DL15, A18, HD3, H162, H193, 12S, 77S, 97S, 106S, and 108S were not well-characterized. Antibodies 3D5, 4E3E, 4G4D, and 11B3AG (gifts of R. N. Lausch) and mAb D10-G12 (from Chiron Corporation, now Novartis) have not been previously described in the literature.

### Virus neutralization assay

The virus neutralization procedure was as previously described with slight modification [75]. Briefly, serial 1:5 dilutions of IgG were mixed with HSV-1 (KOS) or HSV-2 (333) and incubated at 37°C for 1 h. The IgG-virus mixture was then added to monolayers of Vero cells (ATCC #CCL-81) grown in 24-well plates. One hour post-infection, cells were overlaid with DMEM containing 1% carboxymethyl-cellulose and 5% FBS and incubated for an additional 2–3 days. Cells were then fixed with 5% formaldehyde-PBS, stained with crystal violet, and plaques were scored. The starting virus titer was adjusted to result in 100 plaques per well and the results are expressed as highest dilution of IgG that yielded a 50% reduction in the number of plaques. Neutralization assays were done at a minimum two times per antibody. Standard deviations of titers are listed as +/- in Table 1, with the exception of numbers cited from the literature [32] and mAb VID, for which we had a limited quantity.

### MAb binding and competition using the continuous flow microspotter (CFM)/ surface plasmon resonance imaging (SPRi)

Epitope binning experiments of 39 anti-gD mAbs was performed on four soluble glycoproteins [gD1(285t), gD1(306t), gD2(285t), gD2(306t)] using the Wasatch Microfluidics CFM/SPRi system. We used a method described previously [43, 44] with the following modifications. A CFM 2 was used to create a 48-spot microarray of amine-coupled mAbs on a CDM200M sensor chip (Xantec GmbH). Upon docking the printer chip into the SPR imager (IBIS MX96), the chip was blocked with ethanolamine and the system primed with a running buffer of PBS-0.01% Tween 20. Epitope binning was performed in a classical sandwich assay format using 100 nM soluble gD as antigen, 100 nM per mAb as analyte, and 1M glycine pH 2.0 for regeneration. All mAbs were tested in the role of both analyte (in solution) and ligand (on chip) to enable a comprehensive matrix of analyte/ligand mAb paris to be explored. Several mAbs (11S, 12S, 45S, HD1, and HD2) were inactive as ligands so their competitive profiles were determined solely from their performance as analytes. SPRi data were processed in SPRint software and analyzed using Wasatch Microfluidics’ Epitope Binning 2.0 software for heat map generation, sorting, and network plotting. Binary sorting routines were used to organize the heat maps and epitope clusters or “bins” were alternatively viewed as community network plots. For ease of comparison between gD isoforms we generated for such community plots per form. Sensorgrams from the epitope binning study showing the gD capture followed by a buffer analyte instead of a mAb analyte were also separately processed to rank order the kinetic dissociation rates (k_d_ values) of gD from the arrayed mAbs. Following calibration, referencing, and zeroing in SPRint, sensorgrams were imported into Scrubber HT 2.0 (BioLogic LLC) and k_d_ values were fit using a 1:1 binding model.

For binding analysis of gD1 mutants, 0.1μM of soluble gD was injected across the printed mAb array; regeneration conditions were the same as above. This procedure was repeated for each gD mutant tested.

### Peptide binding analysis

To create a peptide array, the entire surface of a CMD200M sensor chip was first coupled with neutravidin in the MX96 imager using a running buffer of 50mM sodium acetate pH 4.5 (coupling buffer) as follows. The surface was activated using a 5 min injection of a 1:1 v/v mixture of 0.4 M 1-Ethyl-3-(3-dimethylaminopropyl)-carbodiimide and 0.1 M sulfo- N-hydroxysuccinimide. Next, 200 μg/ml of neutravidin (diluted in coupling buffer) was injected for 10 min, excess reactive esters were quenched by injecting 0.5 M ethanolamine pH 8.5 for 5 min, and the surface was post-conditioned by injecting 10% glycerol for 1 min. In each case, the MX96 imager uses 100 μL/injection and cycles that volume back and forth across a single flow cell for the time indicated. The neutravidin chip was then loaded into the Wasatch Microfluidics CFM to allow for the parallel capture of 48 biotinylated peptides on individual spots of the chip surface. A synthetic library comprising an overlapping set of 20-mer peptides spanning the gD2 (strain G) ectodomain (residues 1–316) with an N-terminal biotin were purchased from Mimotopes (Australia) [40]. Peptides were prepared to 20 μg/mL in PBS- 0.01% Tween-20 and captured onto individual spots of the neutravidin chip in the CFM for 15 min; the CFM draws 70 μl/print and cycles that volume back and forth across the surface for the indicated time. The printed chip was then redocked into the IBIS MX96 and primed in running buffer of PBS- 0.01% Tween-20. MAbs were diluted to 100 nM in running buffer and injected across the peptide array for 5 min, followed by 2 min of dissociation (running buffer). After each mAb injection, the surface was regenerated by a 30 s injection of 50 mM glycine pH 1.5 followed by a 30 s injection of 100 mM sodium bicarbonate pH 10.

### SPR analysis using the BIAcore 3000

We employed the BIAcore to validate many of the interactions observed using the CFM/IBIS MX96, using conditions previously employed for epitope mapping [75–77]. An anti-His antibody (Qiagen, Inc., Valencia, CA) was anime-coupled to a CM5 sensor chip (GE Healthcare Bio-Sciences, Pittsburgh, PA) following standard procedures. One hundred and fifty resonance units of purified gD2 (285t) or gD2(306t) was captured by the anti-His antibody via its C-terminal His tag. Purified IgGs (100 μg/ml) were then injected for 240 s and the binding was recorded. After each experiment, the chip surface was treated with brief pulses of 0.2 M Na_2_CO_3_ (pH 10) until the RU signal returned to baseline. All injections were performed at a flow rate of 5 μl/min and a running buffer of HBS-EP buffer (10 mM HEPES, pH 7.4, 150 mM NaCl, 3 mM EDTA, 0.005% surfactant P20) was used.

## Supporting information

S1 Fig Updated anti-gD mAb tree (dendrogram). Reorganized anti-gD mAb tree showing relatedness of mAbs through peptide binding, gD mutant binding, and competitive mAb binding analyses. Unlike past versions of our tree, mAbs are not separated according to type specificity. Groups are colored to reflect community mapping as shown in Fig 3. Group XVII is not colored because these mAbs are members of two different communities (green and brown). Dotted boxes surround mAbs that exhibit strong competition across groups. Virus-neutralizing mAbs are colored magenta. Known epitope residues are indicated by numbers; numbers at the top of the group are residues of an epitope shared by all group members, while numbers below a mAb name are specific for that particular mAb. T1S, type-1 specific; T2S, type-2 specific.
(TIF)

S2 Fig Binning of mAbs against gD1(285t). (A) Heat map. For gD1(285t), 36 of our 39 mAbs are represented. Of those mAbs that are missing from the heat map, one is type-2 specific (MC2) and two require residues 286–306 (AP7, 12S). (B) Combined dendrogram.
(TIF)

S3 Fig Binning of mAbs against gD2(306t). (A) Heat map. For gD2(306t), 31 of our 39 mAbs are represented. Of those mAbs that are missing from the heat map, three are type-1 specific (A18, 3D5, 45S) and five bound gD(306t) poorly (≤10 RU) (DL11, 77S, 97S, 106S, 108S). (B) Combined dendrogram.
(TIF)

S4 Fig Binning of mAbs against gD1(306t). (A) Heat map. For gD1(306t), 33 of our 39 mAbs are represented. Of those mAbs that are missing from the heat map, one is type-2 specific (MC2) and five bound gD(306t) poorly (≤10 RU) (DL11, 77S, 97S, 106S, 108S). (B) Combined dendrogram.
(TIF)

S5 Fig Binding map of group I mAbs on gD1 ectodomain mutants. (A) Stick figure representation of full-length gD. The ectodomain is colored light gray, the signal sequence (sig) dark gray, the transmembrane region (TMR) black, and the endodomain white. Amino acid numbers are listed below. (B) Hat map depicting mutant gD binding to printed mAbs via the Wasatch CFM-IBISMX96. MAb names are listed across the top, with groups colored according to Fig 3. The names of soluble gD mutants are listed in the left column. Green, >25 response units (RU) of gD binding to mAb. Yellow, 10–25 RU (low gD binding). Red, <10 RU (no gD binding).
(TIF)
