# Supplementary figures and images for: Global sensing of the antigenic structure of herpes simplex virus gD using high-throughput array-based SPR imaging
Source: PLoS Pathog. 2017 Jun 14;13(6):e1006430. doi: 10.1371/journal.ppat.1006430 (PMC5484518; doi:10.1371/journal.ppat.1006430)

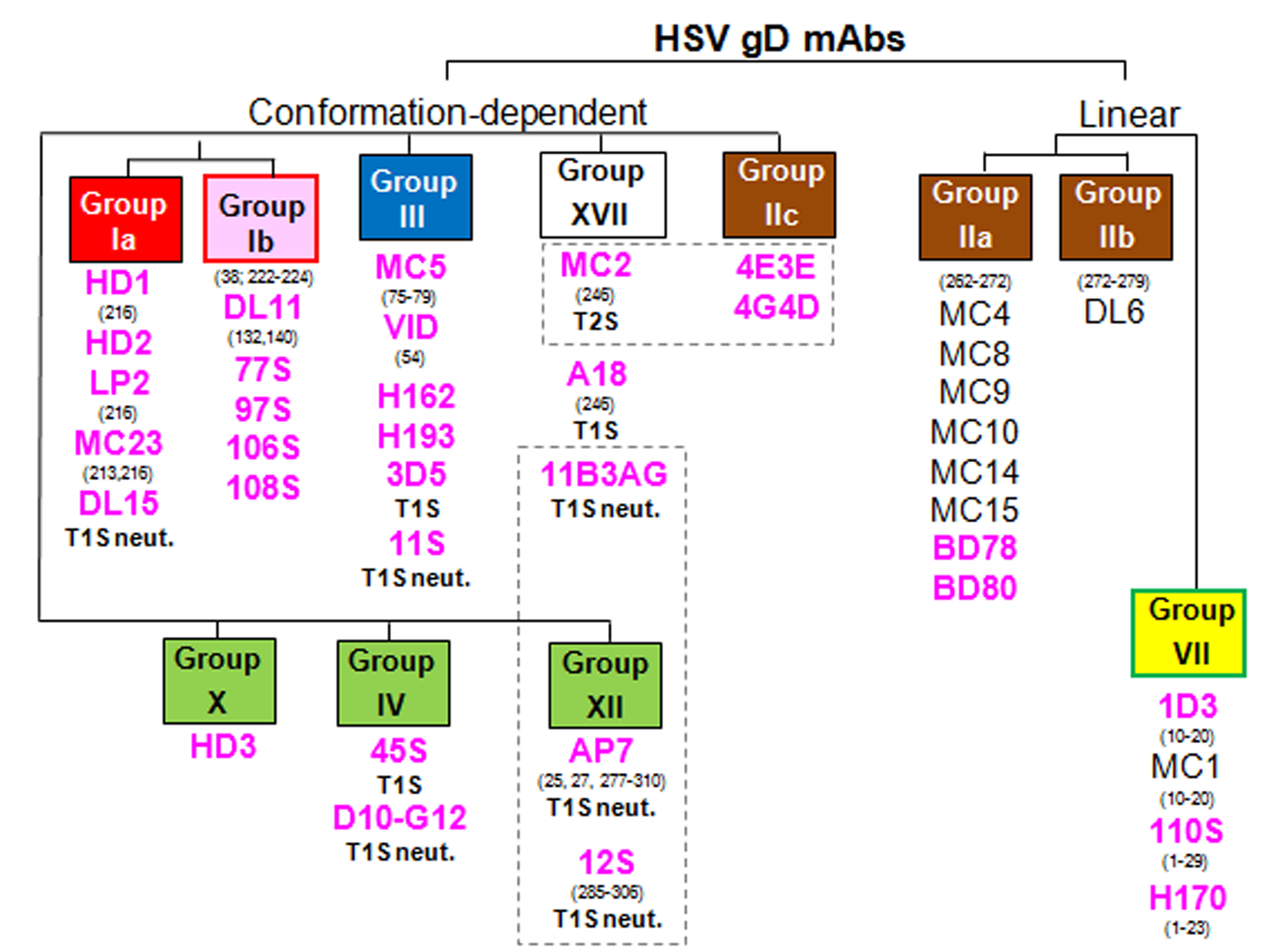

Supplement: S1 Fig — Reorganized anti-gD mAb tree showing relatedness of mAbs through peptide binding, gD mutant binding, and competitive mAb binding analyses. Unlike past versions of our tree, mAbs are not separated according to type specificity. Groups are colored to reflect community mapping as shown in Fig 3. Group XVII is not colored because these mAbs are members of two different communities (green and brown). Dotted boxes surround mAbs that exhibit strong competition across groups. Virus-neutralizing mAbs are colored magenta. Known epitope residues are indicated by numbers; numbers at the top of the group are residues of an epitope shared by all group members, while numbers below a mAb name are specific for that particular mAb. T1S, type-1 specific; T2S, type-2 specific. (TIF) [file ppat.1006430.s001.tif]

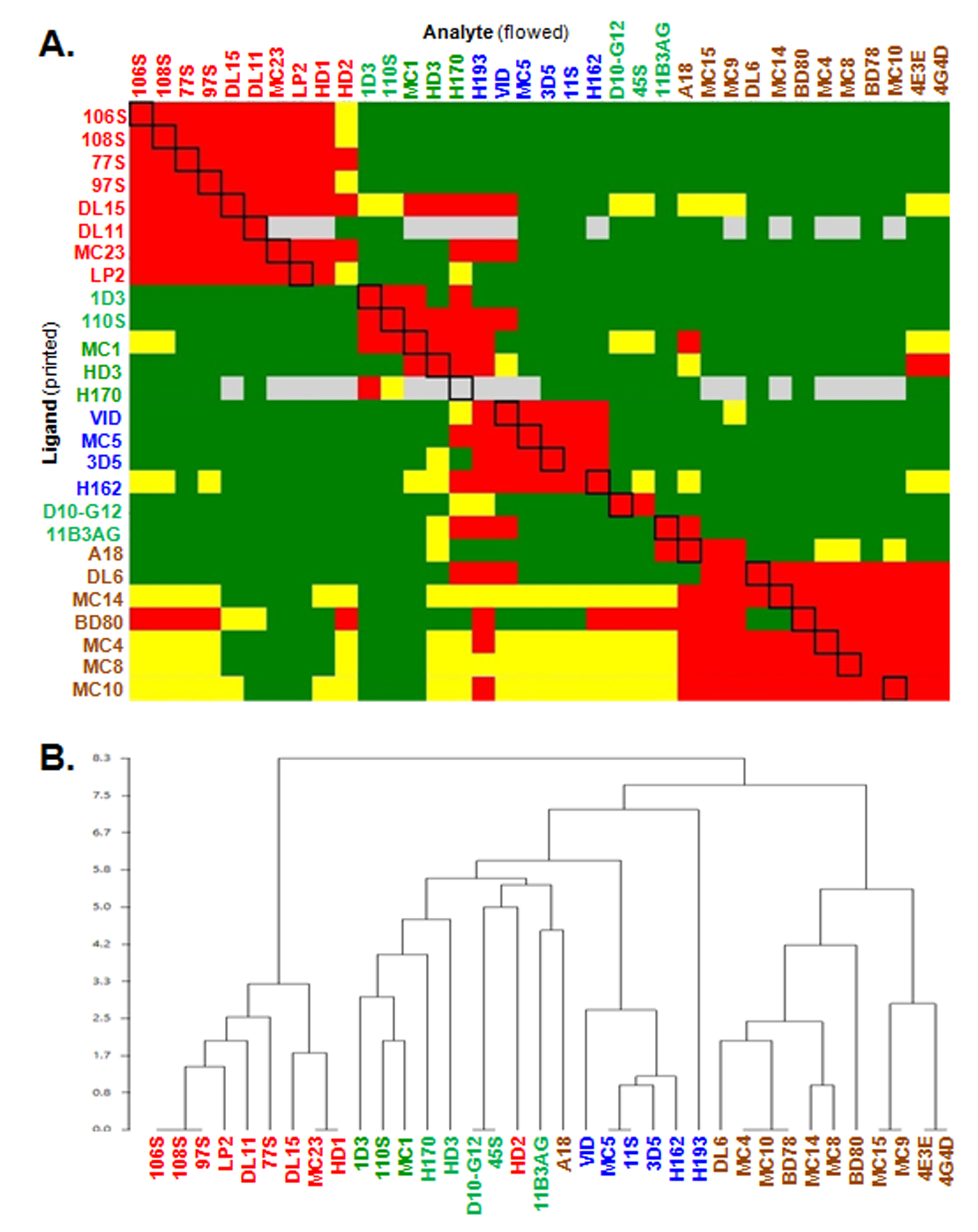

Supplement: S2 Fig — (A) Heat map. For gD1(285t), 36 of our 39 mAbs are represented. Of those mAbs that are missing from the heat map, one is type-2 specific (MC2) and two require residues 286–306 (AP7, 12S). (B) Combined dendrogram. (TIF) [file ppat.1006430.s002.tif]

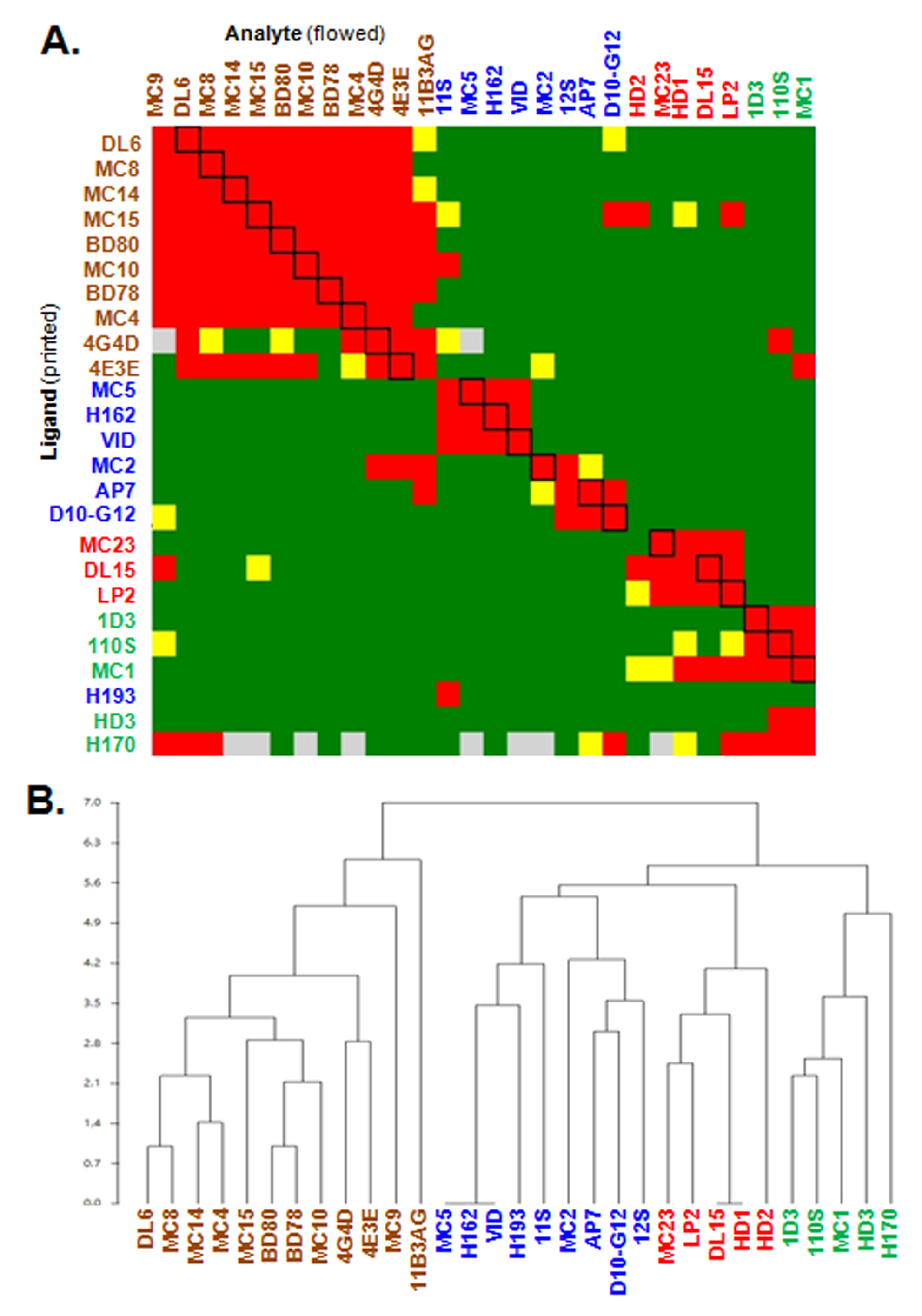

Supplement: S3 Fig — (A) Heat map. For gD2(306t), 31 of our 39 mAbs are represented. Of those mAbs that are missing from the heat map, three are type-1 specific (A18, 3D5, 45S) and five bound gD(306t) poorly (≤10 RU) (DL11, 77S, 97S, 106S, 108S). (B) Combined dendrogram. (TIF) [file ppat.1006430.s003.tif]

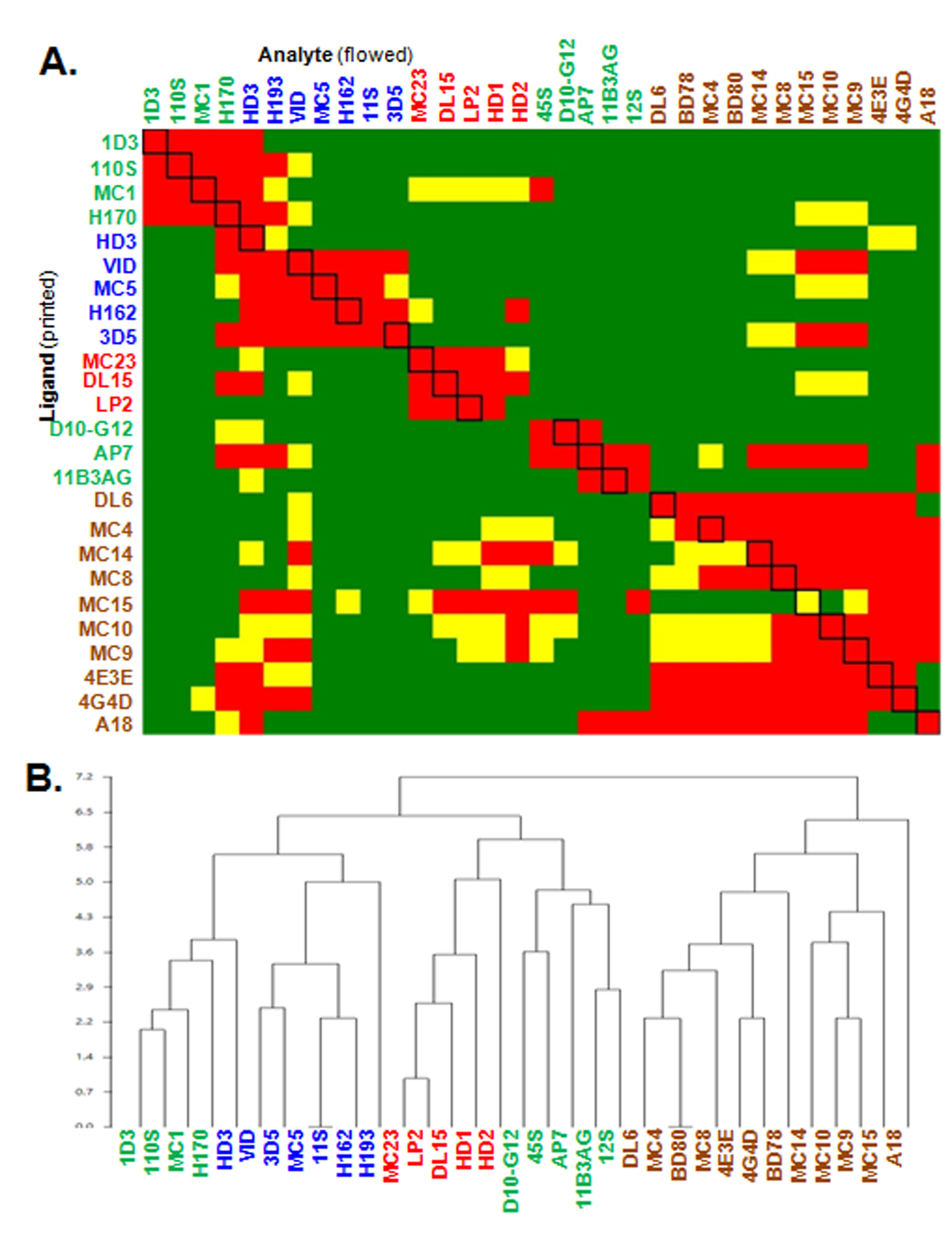

Supplement: S4 Fig — (A) Heat map. For gD1(306t), 33 of our 39 mAbs are represented. Of those mAbs that are missing from the heat map, one is type-2 specific (MC2) and five bound gD(306t) poorly (≤10 RU) (DL11, 77S, 97S, 106S, 108S). (B) Combined dendrogram. (TIF) [file ppat.1006430.s004.tif]

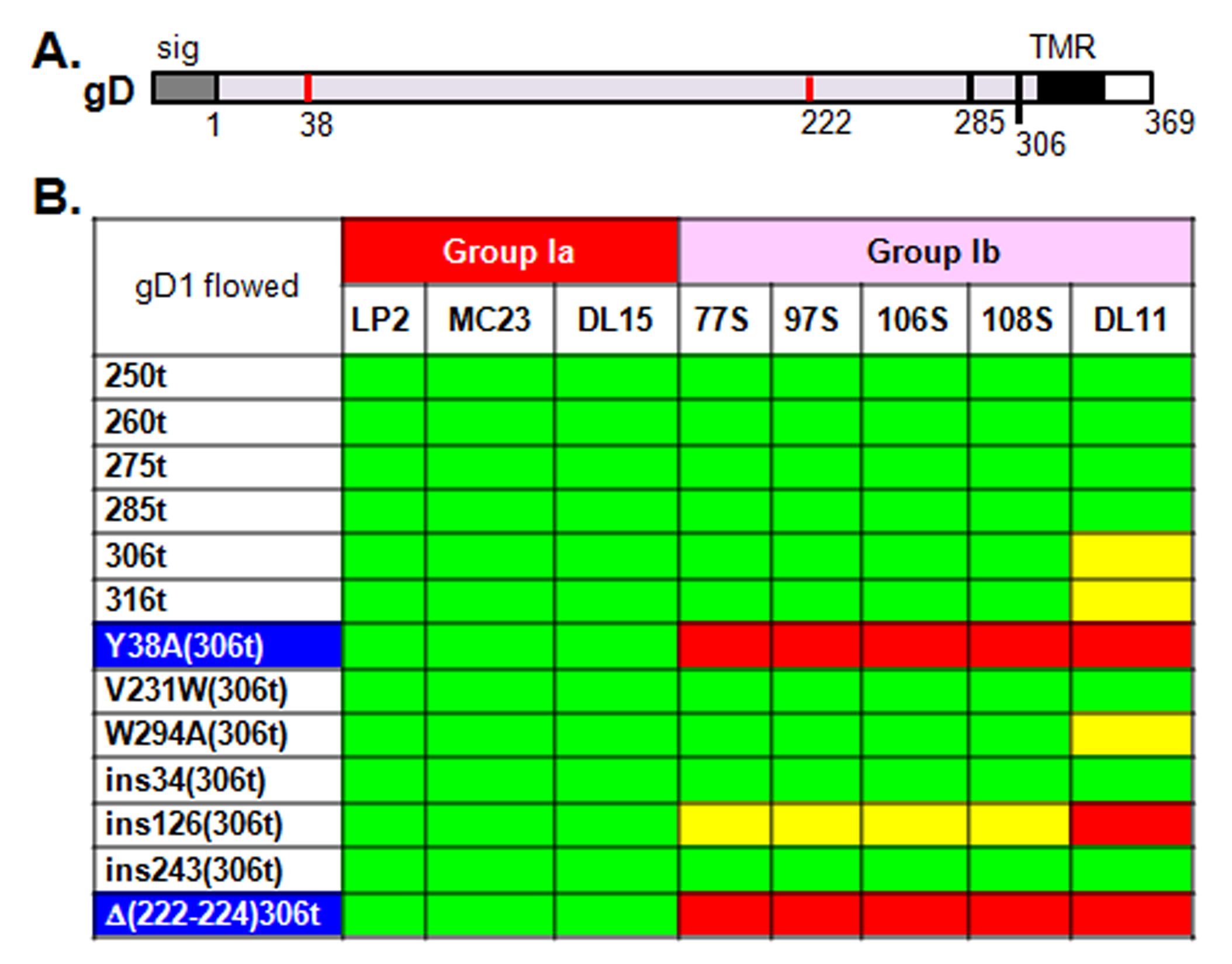

Supplement: S5 Fig — (A) Stick figure representation of full-length gD. The ectodomain is colored light gray, the signal sequence (sig) dark gray, the transmembrane region (TMR) black, and the endodomain white. Amino acid numbers are listed below. (B) Hat map depicting mutant gD binding to printed mAbs via the Wasatch CFM-IBISMX96. MAb names are listed across the top, with groups colored according to Fig 3. The names of soluble gD mutants are listed in the left column. Green, >25 response units (RU) of gD binding to mAb. Yellow, 10–25 RU (low gD binding). Red, <10 RU (no gD binding). (TIF) [file ppat.1006430.s005.tif]
